# Unraveling the Role of MDK‐SDC4 Interaction in Pancreatic Cancer‐Associated New‐Onset Diabetes by Single‐Cell Transcriptomic Analysis

**DOI:** 10.1002/advs.202409987

**Published:** 2025-07-25

**Authors:** Zengyu Feng, Jianyao Lou, Chuanshuai Lin, Haocheng Yu, Yuheng Tu, Jiali Gong, Xiawei Li, Yulian Wu

**Affiliations:** ^1^ Department of General Surgery Second Affiliated Hospital Zhejiang University School of Medicine Hangzhou Zhejiang 310009 China; ^2^ Key Laboratory of Cancer Prevention and Intervention China National Ministry of Education Cancer Institute Second Affiliated Hospital Zhejiang University School of Medicine Hangzhou Zhejiang 310009 China; ^3^ Cancer Center Zhejiang University Hangzhou Zhejiang 310009 China; ^4^ Department of Surgery Fourth Affiliated Hospital Zhejiang University School of Medicine Yiwu Zhejiang 322000 China

**Keywords:** MDK‐SDC4 interaction, pancreatic cancer‐associated new‐onset diabetes, Ras signaling pathway, single cell RNA‐sequencing, SP1

## Abstract

Elevated blood glucose levels may serve as an early indicator of underlying pancreatic cancer. Discriminating between pancreatic cancer‐associated new‐onset diabetes (PCAND) and new‐onset type 2 diabetes mellitus (T2DM) holds promise for enabling an earlier diagnosis of pancreatic cancer. Nevertheless, the absence of effective biomarkers for distinguishing PCAND from the more prevalent new‐onset T2DM persists, primarily because of the elusive pathogenesis of PCAND. In this study, the intricate intercellular communication is comprehensively elucidated through single‐cell RNA sequencing. The findings identified Midkine (MDK) as a potential mediator of the interaction between tumor and beta cells. MDK, which originated from pancreatic ductal adenocarcinoma cells, exerted deleterious effects on paraneoplastic beta cells by binding to the SDC4 receptor on the beta cell surface and subsequently downregulating the Ras signaling pathway, thereby impairing insulin production and secretion. Notably, the plasma levels of MDK are higher in patients with PCAND than in those with T2DM. In conclusion, MDK has emerged as a pivotal driver of PCAND pathogenesis and may function as a blood‐based biomarker for discriminating between PCAND and T2DM in populations with new‐onset diabetes, thereby facilitating the advancement of early detection strategies for pancreatic cancer.

## Graphical abstract

Midkine (MDK) is a mediator of the interaction between pancreatic cancer and beta cells. MDK, which originated from pancreatic ductal adenocarcinoma cells, exerted deleterious effects on paraneoplastic beta cells by binding to the SDC4 receptor on the beta cell surface and subsequently downregulating the Ras signaling pathway, thereby impairing insulin production and secretion.

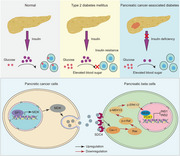

## 1 Introduction

Pancreatic ductal adenocarcinoma (PDAC) accounts for 90% of all pancreatic cancers and presents a substantial challenge in clinical management because of its stealthy onset, rapid progression, and high mortality rate.^[^ ^1^, ^2^, ^3^ ^]^ The formidable lethality of this disease is principally attributed to the fact that more than 50% of patients are diagnosed at a late stage^[^ ^4^ ^]^ and only 10% of individuals are viable candidates for surgical resection.^[^ ^5^ ^]^ Early detection is crucial for enhancing patient survival;^[^ ^6^ ^]^ nevertheless, whole population‐based screening is impractical and economically unfeasible because of the relatively low incidence of PDAC (13.1/100 000 individuals^[^ ^7^ ^]^). The US Preventive Services Task Force (USPSTF) does not advocate PDAC screening in the general population.^[^ ^8^ ^]^ Conversely, the USPSTF recommends PDAC screening for high‐risk individuals, such as those with inherited genetic syndromes, obesity, and new‐onset diabetes.^[^ ^9^ ^]^

Patients with new‐onset diabetes occurring after 50 years of age have been identified as a high‐risk population susceptible to PDAC.^[^ ^10^, ^11^, ^12^ ^]^ Predominantly, 90–95% of individuals within this demographic are diagnosed with type 2 diabetes mellitus (T2DM), whereas only 1% are diagnosed with pancreatic cancer‐associated new‐onset diabetes (PCAND), a subtype of type 3c diabetes mellitus (T3cDM).^[^ ^13^, ^14^, ^15^ ^]^ Compared to the general population, patients with T2DM present a moderately elevated risk of developing PDAC (2.05–3.05‐fold), while PCAND is associated with a notably heightened risk (4.18–9.78‐fold).^[^ ^16^ ^]^ Two independent Mendelian randomization studies investigating the associations among T2DM, PCAND, and PDAC have consistently demonstrated a causal relationship between PCAND and PDAC, whereas T2DM is not causally linked to PDAC.^[^ ^16^, ^17^ ^]^ On average, individuals afflicted with PDAC manifest hyperglycemia at 30–36 months prior to tumor diagnosis, presenting a potential window for early PDAC detection.^[^ ^18^ ^]^ These observational findings underscore the substantial risk of PDAC in PCAND populations and suggest practical screening strategies based on this phenomenon to effectively capture sporadic and asymptomatic PDAC cases. Hence, precise differentiation between PCAND and new‐onset T2DM, which is more prevalent, has significant implications for PDAC screening.

Differentiating between PCAND and new‐onset T2DM remains a challenge.^[^ ^19^ ^]^ Traditional risk factors for diabetes, such as body mass index, and clinical characteristics, including fasting blood glucose levels, fail to yield valuable insights for effective distinction.^[^ ^20^, ^21^, ^22^ ^]^ Recently, several serum biomarkers associated with PCAND that exhibit excellent diagnostic performance have been identified.^[^ ^23^, ^24^, ^25^, ^26^ ^]^ However, their expression patterns have primarily been assessed in PCAND and PDAC without concurrent diabetes, raising the question as to whether these biomarkers can effectively distinguish PCAND from T2DM. Notably, a 6‐serum microRNA panel,^[^ ^27^ ^]^ a 58‐plasma metabolite signature,^[^ ^28^ ^]^ and an 8‐clinical parameter model^[^ ^29^ ^]^ have emerged as potential biomarkers for the precise discrimination between PCAND and new‐onset T2DM. Nonetheless, the inclusion of excessive factors and the low abundance of certain elements may increase the cost and complexity of detection, thereby limiting the widespread applicability of these models in clinical practice. Balasenthil et al. recently proposed a blood‐based migration signature biomarker panel that demonstrated moderate accuracy in discriminating between PCAND and T2DM.^[^ ^30^ ^]^ However, this signature is based on two secreted proteins that display differential expression between pancreatic tumors and normal samples, lack specificity as mediators of PCAND, and possess limited biological relevance to PCAND. In conclusion, there is a dearth of acceptable biomarkers to effectively distinguish PCAND from T2DM.

The pathological mechanisms underlying PCAND and T2DM are distinct,^[^ ^31^ ^]^ and the identification of diabetogenic tumor‐specific products may pave the way for significant advancements in the development of effective biomarkers. Diabetes is reportedly present in over 50% of individuals with PDAC,^[^ ^32^ ^]^ and 57% of PCAND cases resolve after tumor removal.^[^ ^20^ ^]^ These clinical findings suggest that PCAND is a paraneoplastic phenomenon induced by injurious products derived from PDAC cells, which subsequently dysregulate glucose homeostasis. Furthermore, recent retrospective studies have indicated that PCAND is primarily characterized by beta cell dysfunction, resulting in inadequate insulin secretion capacity.^[^ ^33^ ^]^ While insulin resistance is also observed in PCAND,^[^ ^34^ ^]^ it is not as pronounced as that observed in T2DM.^[^ ^33^ ^]^ Therefore, elucidating the pathogenesis of PCAND and identifying the key mediators responsible for paraneoplastic beta cell dysfunction are important for targeted screening of PDAC within the PCAND population.

Data regarding the communication between PDAC cells and paraneoplastic beta cells are scarce. While previous reports have demonstrated the inhibitory effect of PDAC cell‐derived exosomes on insulin secretion,^[^ ^35^, ^36^, ^37^ ^]^ the specific secreted proteins involved in the interactions between cancer cells and beta cells have not yet been comprehensively explored. Single‐cell RNA sequencing (scRNA‐seq) has emerged as a robust platform for unraveling the intricate tumor microenvironment and for providing a comprehensive overview of the interactions among diverse cell types.^[^ ^38^, ^39^, ^40^ ^]^ To date, no studies have leveraged scRNA‐seq analysis to characterize cell‐cell interactions relevant to the pathogenesis of PCAND.

In the current study, we compiled publicly available scRNA‐seq data from normal human islets, non‐malignant pancreatic tissues, and pancreatic tumor tissues to construct a comprehensive pancreatic atlas. We delineated intercellular communication networks and uncovered a novel interaction between tumor and beta cells mediated by Midkine (MDK) and Syndecan‐4 (SDC4). We found that MDK was transcriptionally activated by Specificity protein 1 (SP1) in PDAC cells and that the MDK‐SDC4 interplay impaired insulin synthesis and secretion by inhibiting the Ras signaling pathway in beta cells. Importantly, elevated plasma MDK levels were detected in patients with PCAND compared with those with T2DM. Our study offers a new perspective for understanding the pathogenesis of PCAND and affirms that serum MDK is a potential biomarker for discriminating PCAND from T2DM.

## 2 Materials and Methods

### 2.1 Datasets

scRNA‐seq data from 24 patients with PDAC and 11 patients without PDAC were obtained from the NGDC database under accession number CRA001160.^[^ ^41^ ^]^ Among 24 patients with PDAC, 10 presented with diabetes while 14 were non‐diabetic. The interval between diabetes diagnosis and PDAC detection was unavailable for patients with comorbid diabetes. Additionally, scRNA‐seq data from 14 normal human islets were sourced from the Gene Expression Omnibus (GEO) database under accession number GSE183568.^[^ ^42^ ^]^ Bulk RNA sequencing data from patients with PDAC, including GSE15471, GSE16515, GSE21501, GSE28735, GSE32676, GSE41368, GSE60979, GSE62165, GSE62452, GSE71729, GSE71989, and GSE183795, were retrieved from the GEO database. Furthermore, the gene expression data of the MTAB‐6134 and MTAB‐6690 cohorts were obtained from the ArrayExpress database, whereas those of the PACA‐CA cohort were sourced from the ICGC database. Gene expression profiles of peripheral blood samples from eight PDAC patients without diabetes and eight PDAC patients with diabetes were downloaded from the GEO database under accession number GSE15932. Gene expression profiles of the PDAC cell lines were acquired from the GEO database under accession numbers GSE138437 and GSE166165. Bulk RNA sequencing data from human pancreatic islets of 39 T2DM and 35 T3cDM donors were obtained from the GEO database under accession number GSE164416.^[^ ^43^ ^]^ All sequencing and microarray data utilized in this study are publicly available and summarized in Table S1 (Supporting Information).

### 2.2 scRNA‐seq Data Preprocessing

Analysis of scRNA‐seq data and generation of graphical representations were conducted using R software version 4.2.0. scRNA‐seq data acquired from normal islets, non‐PDAC patients, and PDAC patients were processed using the Harmony package (version 0.1.1) to eliminate batch effects and integrate all samples. The Harmony package was applied with default settings, except for the theta parameter (set to 2) to balance batch correction against biological signal retention. Batch labels were explicitly provided as metadata during integration. Batch correction efficacy was evaluated by visualizing the integrated embedding using t‐distributed stochastic neighbor embedding (t‐SNE), confirming enhanced inter‐sample mixing relative to the uncorrected analysis. Subsequently, the Seurat package (version 4.3.0) was used for data refinement, involving the removal of low‐quality cells based on standard criteria (<300 genes/cell and <3 cells/gene). Subsequently, 2000 highly variable genes (HVGs) were selected for analysis. Dimensionality reduction and unsupervised clustering of the scRNA‐seq data were performed using default parameters within the Seurat package. Subsequent cell annotation was based on widely recognized marker genes. The outcomes of cell clustering and annotation were visualized using the t‐SNE function, and the differential genes for distinct subgroups were identified using the FindMarkers and FindAllMarkers functions.

### 2.3 Cell–Cell Communication Analysis

Quantitative characterization of cellular communication networks was performed using the CellChat package (version 1.1.3), a powerful platform known for its accurate inference of intercellular connections through the computation of ligand‐receptor interactions.^[^ ^44^ ^]^ By employing default parameters and the ligand‐receptor pair database, the quantity and strength of communication between distinct cell clusters were computed. These intercellular contacts were further aggregated to construct communication networks separately for PDAC and non‐PDAC samples. Of particular interest in our study were the potential interactions between PDAC cells and beta cells. Consequently, we compared the communication networks of tumor samples with those of non‐tumor samples to identify distinctive interaction patterns under disease conditions. The results were visualized using the CellChat and ggplot2 packages (version 3.4.2).

### 2.4 Copy Number Variation (CNV) Analysis

CNV patterns of single cells were estimated using the inferCNV package (version 1.14.2). The single‐cell gene expression matrix extracted from Seurat was normalized, standardized, and used as an input to calculate CNV scores for different cell clusters using default parameters. T cells and endothelial cells were used as controls. Typically, malignant cells exhibit elevated CNV scores.

### 2.5 Functional Enrichment Analysis

Functional annotation and pathway enrichment analyses were performed using clusterProfiler (version 4.7.1). Differentially expressed genes (DEGs) or cluster‐specific genes obtained from FindMarkers or FindAllMarkers in Seurat were subjected to Gene Ontology (GO) analysis. Significantly correlated (*p* < 0.05) biological processes were selected for visualization. Gene set enrichment analysis (GSEA) was performed using the clusterProfiler and msigdbr (version 7.5.1) packages. The target gene sets were acquired using the msigdbr package. Hallmark pathways with an adjusted P‐value of >0.05 were excluded from visualization.

### 2.6 Pseudotime Trajectory Analysis

To investigate the differentiation trajectory of ductal cells, we used CytoTRACE (version 0.3.3), Slingshot (version 2.6.0), and Monocle2 (version 2.18.0) packages with default parameters. The single‐cell gene expression data generated by Seurat served as the input for the CytoTRACE function to compute the CytoTRACE scores for different cell clusters. For pseudo‐time trajectory analysis, the gene expression matrix was subjected to a monocle algorithm to establish a new CellDataSet object. The differentiation trajectory and pseudo‐time of the ductal cell populations were inferred following gene filtering, dimension reduction, and cell ordering. Outcomes were visualized using the CytoTRACE, Monocle2, and ggplot2 packages.

### 2.7 Clinical Tissue and Plasma Samples

A total of 88 PDAC specimens were collected from patients diagnosed and treated with surgical pancreatectomy, with pathological confirmation at the Second Affiliated Hospital of Zhejiang University School of Medicine. Corresponding follow‐up data were available for 70 PDAC patients and used for survival analysis. Tissues were embedded in paraffin and subjected to immunohistochemical staining. Plasma samples were obtained from healthy controls (N = 6), intraductal papillary mucinous neoplasm (IPMN, N = 6), T2DM (N = 13), PDAC without diabetes (N = 20), PDAC with long‐standing (diagnosed > 3 years prior to PDAC diagnosis) T2DM (PCLSD, N = 12) and PCAND (diagnosed ≤ 2 years prior to PDAC diagnosis; N = 11). All patients were treatment‐naïve before sample collection; detailed clinical characteristics were provided in Table S2 (Supporting Information). Student's *t*‐test analysis was used for comparison between two groups. The diagnostic criteria for diabetes were in accordance with American Diabetes Association.^[^ ^45^ ^]^ Plasma MDK levels in various groups were measured using a human MDK enzyme‐linked immunosorbent assay (ELISA) kit (CUSABIO, Wuhan, China) following the manufacturer's instructions. Prior to enrollment, written informed consent was obtained from all patients. The Ethics Committee of the Second Affiliated Hospital of Zhejiang University School of Medicine approved this study.

### 2.8 Cells and Cell Culture

Human PDAC cell lines (BxPC‐3, CFPAC‐1, MIAPaCa‐2, and PANC‐1), mouse pancreatic insulinoma cells (beta‐TC‐6), and human embryonic kidney cells (HEK293T) were obtained from the Chinese Academy of Sciences’ Committee of Type Culture Collection (Shanghai, China). Mouse pancreatic insulinoma (MIN6) cells were obtained from Beina Chuanglian Biotechnology (Beijing, China). Human pancreatic ductal immortalized cells (hTERT‐HPNE), human PDAC cell line PaTu‐8988T, mouse pancreatic alpha cells (alpha TC1 clone 6), and mouse PDAC cell line PANC02 were purchased from Pricella Biotechnology (Wuhan, China). The cells were cultured in the following specific media: BxPC‐3 cells in RPMI 1640 medium (Sigma); CFPAC‐1 cells in IMDM medium (Sigma); alpha TC1 cells in MEM medium (Sigma); and PANC‐1, MIA PaCa‐2, beta‐TC‐6, HPNE, PaTu‐8988T, PANC02, and HEK‐293T cells in high‐glucose DMEM (Sigma). Beta‐TC‐6 medium was supplemented with 20% fetal bovine serum (FBS; VivaCell), whereas all other media were supplemented with 10% FBS. All media were supplemented with 100 U mL^−1^ penicillin and streptomycin (Cienry, Zhejiang, China). MIN6 cells were cultured in RPMI 1640 medium, 10% FBS, and 100 µm β‐mercaptoethanol (Sigma). All cells were maintained at 37 °C with 5% CO2, authenticated by STR profiling, and tested negative for mycoplasma contamination.

### 2.9 Plasmid Construction and Cell Transfection

For knockdown assays, short hairpin RNAs (shRNAs) targeting human MDK, mouse MDK, mouse NCL, mouse NOTCH2, mouse SDC4, mouse Cav‐1, and human SP1 were individually cloned into the CMV‐copGFP‐T2A‐puro‐H1‐shRNA vector (BioeGene, Shanghai, China). The specific shRNA sequences are listed in Table S3 (Supporting Information). Mouse SDC4 cDNA were synthesized and subcloned into a pCDH‐CMV (CMV‐MCS‐3FLAG‐EF1a‐coGFP‐P2A‐PURO) lentivirus vector (BioeGene). In dual‐luciferase reporter assays, sequences of the potential SP1 binding site on the MDK promoter and its corresponding mutants were inserted into the pGL3 luciferase reporter vector (BioeGene). For transient transfection, plasmids were co‐transfected into human HEK–293T cells using Lipofectamine 2000 (Invitrogen), following the manufacturer's instructions. In the case of stable transfection, packaging plasmid psPAX2, envelope plasmid pMD2.G, and targeting plasmids were co‐transfected, then filtered through a 0.45‐µm‐diameter pore (Millipore, USA), and added to the cell culture medium supplemented with polybrene (Yeasen, Shanghai, China). Stably transfected cells were selected using puromycin (Beyotime).

### 2.10 RNA Extraction and Real‑Time Quantitative polymerase Chain Reaction (RT‐qPCR)

Total RNA was extracted using the FastPure Cell/Tissue Total RNA Isolation Kit V2 (Vazyme, Nanjing, China) according to the manufacturer's instructions. Subsequently, cDNA was synthesized from 1 µg of purified total RNA utilizing the HiScript III 1st Strand cDNA Synthesis Kit (+gDNA wiper) (Vazyme). Quantitative PCR was carried out using the ChamQ Universal SYBR qPCR Master Mix (Vazyme) in the LightCycler 480 II real‐time PCR detection system (Roche, Switzerland). For mouse genes, β‐actin was employed as an internal control; for human genes, glyceraldehyde‐3‐phosphate dehydrogenase (GAPDH) was used. The expression levels of the target genes were determined using the 2‐ΔCT method. The primer sequences used for RT‐qPCR are listed in Table S4 (Supporting Information).

### 2.11 Western Blotting

The cells were rinsed twice with phosphate buffered saline (PBS) and lysed in 1 × Omni‐Easy protein sample loading buffer (EpiZyme, Shanghai, China). Subsequently, the cell lysates were separated on 12.5% polyacrylamide gel electrophoresis gels (PG213, EpiZyme) and transferred to polyvinylidene fluoride membranes (Millipore). Subsequently, the membranes were blocked with PBST containing 5% bovine serum albumin (BSA, Beyotime) for 1 h, followed by incubation with primary and secondary antibodies. Details of the antibodies used for western blotting are shown in Table S5 (Supporting Information).

### 2.12 Immunofluorescence (IF) Staining

The beta‐TC‐6 and MIN6 cells were seeded on 24‐well coverslips at ≈50% confluence and pretreated with control medium and conditioned medium for 24 h for insulin staining. Following this, the cells were fixed with 4% paraformaldehyde at 4 °C overnight, rinsed with PBS for 5 min three times, permeabilized using a blocking buffer (0.1% Triton‐X100 and 3% BSA in PBS) at room temperature for 30 min, and subsequently incubated with primary antibodies at 4 °C overnight. IF staining was performed using anti‐insulin antibody (1:200 dilution, ab181547, Abcam), anti‐MDK antibody (1:100 dilution, 11009‐1‐AP, Proteintech), and anti‐SDC4 antibody (200 µg mL^−1^, sc‐12766, Santa Cruz Biotechnology). Following primary antibody incubation, the cells were washed three times with PBS for 5 min and then incubated with secondary antibodies conjugated with Alexa Fluor 488 (1:200 dilution, 4416S, CST) or Alexa Fluor 555 (1:200 dilution, 4409S, CST) for 1 h, while avoiding exposure to light. Subsequently, the cells were washed thrice with PBS for 5 min and stained with DAPI (1:1000 dilution, Beyotime) at room temperature for 5 min. Finally, the cells were imaged and observed under a Leica Stellaris 5 confocal microscope.

### 2.13 Immunohistochemistry (IHC) Staining

IHC assays were conducted on paraffin sections obtained from human PDAC tissues and mouse pancreas. Initially, the sections were deparaffinized in xylene, rehydrated through an alcohol gradient, and subjected to antigen retrieval by heating for 15 min in citrate buffer (pH 6.0). Subsequently, they were immersed in 3% hydrogen peroxide for 25 min to inhibit endogenous peroxidase activity, followed by blocking for nonspecific binding using 5% goat serum, and then incubated with antibodies at 4 °C overnight. The primary antibodies used for IHC assays were MDK (1:100 dilution, ab52637, Abcam), SP1 (1:2000 dilution, ab231778, Abcam), and insulin (1:30 000 dilution, ab181547, Abcam). Following primary antibody incubation, the sections were incubated with HRP‐conjugated secondary antibodies, treated with 3,3‐diaminobenzidine solution, counterstained with hematoxylin, scanned using a Pannoramic MIDI scanner (3DHISTECH, Hungary), and visualized using SlideViewer software (3DHISTECH). The expression levels of MDK and SP1 were determined based on the intensity of immunostaining (0 = negative, 1 = weak, 2 = moderate, 3 = strong) and percentage (0 = 0%, 1 = 1–25%, 2 = 26–50%, 3 = 51–75%, 4 = 76–100%). These two scores were multiplied to derive the final IHC scores, which ranged from 0 to 12. For survival analysis, IHC scores <50% were classified as low expression, whereas scores >50% were classified as high expression. Regions of interest (ROIs) where pancreatic tumor tissues were immediately adjacent to pancreatic islet tissues were identified in 57 PDAC tissue sections. IHC scoring for both MDK and insulin was performed on these ROIs. For patients with multiple qualifying ROIs, the average score was calculated per patient. Non‐integer average scores were rounded to the nearest integer.

### 2.14 Conditioned Medium Collection

After 12 h of cell attachment, the growth medium of transfected or wild‐type PDAC cells was replaced with RPMI‐1640 devoid of FBS. Subsequently, after 48 h of incubation, the medium was collected and subjected to centrifugation at 3000 rpm for 15 min at 4 °C to eliminate non‐viable cells. The resultant supernatants, termed as conditioned medium, were preserved at −80 °C and utilized for experiments within 2 weeks. Prior to their application in the treatment of beta‐TC‐6 or MIN6 cells, both conditioned medium and fresh RPMI‐1640 (termed control medium) were supplemented with 10% FBS. The cells were maintained in these media for 48 h, with the medium renewed every 24 h, after which they were harvested for subsequent experiments.

### 2.15 Glucose‐Stimulated Insulin Secretion (GSIS)

For the GSIS assay, beta‐TC‐6 (5 × 10^4^ cells/well) and MIN6 (1 × 10^5^ cells/well) cells were seeded into 24‐well plates and cultured in either control medium or conditioned medium. Subsequent to 48 h of incubation, the cells underwent a single wash with Krebs‐Ringer bicarbonate (KRB) buffer (constituted of 114 mm NaCl, 2.5 mm CaCl2, 4.7 mm KCl, 1.16 mm MgSO4, 1.2 mm KH2PO4, 25.5 mm NaHCO3, 20 mm HEPES, 0.2% BSA, pH 7.4). Following this, the cells were pre‐incubated in KRB buffer devoid of glucose for 1 h at 37 °C. Post pre‐incubation, the buffer was replaced with KRB buffer containing either 2.8 mm glucose (designated as low glucose) or 16.8 mm glucose (designated as high glucose) for 1 h at 37 °C. Subsequently, the supernatants were individually collected and subjected to centrifugation at 1000 × g for 15 min at 4 °C. Insulin levels in distinct supernatants were determined using the Mouse Insulin ELISA Kit (ab277390, Abcam) in accordance with the manufacturer's instructions.

### 2.16 In Vivo Assays

To establish in vivo PCAND models, both subcutaneous and orthotopic injections of PDAC cells were employed. A schematic of the subcutaneous model experimental design is presented in Figure 6A. Briefly, stable MDK knockdown (shMDK#1 and shMDK#2) and control (shNC) CFPAC‐1 cells were subcutaneously injected (4 × 10^6^ cells/100 µL PBS) into the flanks of recipient nude mice (Vital River, Beijing, China). Similarly, stable MDK knockdown and control PANC02 cells were subcutaneously injected (5 × 10^6^ cells/100 µL PBS) into the flanks of recipient C57BL/6 mice (Vital River). For the orthotopic model, luciferase‐expressing CFPAC‐1 cells with or without MDK knockdown (1 × 10⁶ cells in 25 µL PBS) were injected orthotopically. Four weeks post‐injection, mice were intraperitoneally administered D‐luciferin potassium salt (150 mg kg^−1^; Beyotime) and imaged using an IVIS Spectrum Imaging System (PerkinElmer, USA).To monitor fasting blood glucose levels, the mice underwent a 6‐h fasting period before blood sampling, and their blood glucose levels were measured using a glucometer (Yuwell, Shanghai, China) every 4 days after the first injection. After a span of four weeks, mice underwent a 24‐h fasting period and were subsequently sacrificed to obtain peripheral blood samples, tumors, and pancreases. Serum was acquired by centrifuging peripheral blood at 3000 rpm for 15 min and was utilized to evaluate fasting insulin levels, which were assessed using the Mouse Insulin ELISA Kit (Abcam). Tumors were weighed, and the pancreases were used for IHC staining, as previously described. All procedures related to animal studies were approved by the Ethics Committee of the Second Affiliated Hospital of Zhejiang University School of Medicine.

### 2.17 RNA Sequencing (RNA‐seq)

High‐throughput RNA‐seq of beta‐TC‐6 cells, both treated with and without recombinant mouse MDK protein (HY‐P79419, MedChemExpress), was performed by LC‐Bio Technologies (Hangzhou, China). Total RNA was extracted and quantified, and its integrity was assessed using a Bioanalyzer 2100 (Agilent Technologies) and denaturing agarose gel electrophoresis. Subsequently, Poly (A) RNA was isolated from 1 µg of total RNA using Dynabeads Oligo(dT) (Thermo Fisher), followed by fragmentation into small segments at elevated temperatures employing the Magnesium RNA Fragmentation Module (NEB, USA). The RNA fragments were then reverse transcribed into cDNA. The synthesis of U‐labeled second‐stranded DNAs involved the utilization of cDNA, E. coli DNA polymerase I (NEB), RNase H (NEB), and a dUTP solution (Thermo Fisher). Following this, bases were added to the blunt ends of each strand, rendering them amenable to connect to indexed adapters that encompassed T bases for ligation. After ligation, the U‐labeled second‐stranded DNAs was treated with heat‐labile UDG enzyme (NEB), and the ligated products were amplified using PCR to construct the cDNA library. Finally, the constructed cDNA library was subjected to 2 × 150 bp paired‐end sequencing (PE150) on an Illumina NovaSeq 6000 instrument, in accordance with the recommended protocol provided by the vendor.

### 2.18 RNA‐seq Data Analysis

The paired‐end reads obtained from the Illumina NovaSeq 6000 sequencer were preprocessed using FASTP software to eliminate contaminated reads and low‐quality bases. Subsequently, high‐quality clean reads were mapped to the reference genome of Mus musculus GRCm38 using the HISAT2 software. The mapped reads were then assembled using StringTie software, and the resulting transcriptomes for each sample were merged using GffCompare software. Following generation of the final transcriptome, StringTie software was used to generate mRNA expression profiles by computing FPKM (fragments per kilobase of transcript per million mapped reads). Differential expression analysis was executed based on FPKM values, identifying mRNAs with fold change >2 and P‐value <0.05 using the R package edgeR. Differentially expressed mRNAs were subsequently subjected to Gene Ontology analysis using the DAVID online platform. To discern distinct gene sets between the two groups, GSEA was performed using GSEA software (version 4.1.0) and MSigDB. The gene expression matrix was input and genes were ranked using the Signal2Noise normalization method. Enrichment scores and P‐values were computed using the default parameters.

### 2.19 Co‐Immunoprecipitation (co‐IP)

For the co‐IP assay, beta‐TC‐6 cells were lysed using RIPA lysis buffer (50 mm Tris‐Base, 150 mm NaCl, 5 mm EDTA, 1% NP‐40, 0.1% SDS, pH 7.4). The cell lysates were then incubated with Anti‐Flag Affinity Gel (HY‐K0217, MedChemExpress) overnight at 4 °C. Following incubation, the immunocomplexes were washed six times with RIPA lysis buffer and mixed with loading buffer for western blotting analysis.

### 2.20 Dual‐Luciferase Reporter Assays

Two days subsequent to the transfection of target plasmids, HEK293T cells were lysed, and the lysates were utilized to determine the activities of Firefly and Renilla luciferase via using the tDual‐Lumi II Luciferase Reporter Gene Assay Kit (Beyotime) according to the manufacturer's instructions. A microplate luminometer (GloMax Discover, Promega) was used to quantify the luciferase activity. Relative luciferase activity was calculated as the ratio of Firefly luciferase to Renilla luciferase activity.

### 2.21 Bioinformatic Analysis of Public Databases

The gene expression profiles of MDK and SP1 in pancreatic adenocarcinoma (PAAD) and normal pancreas were acquired from the Gene Expression Profiling Interactive Analysis (GEPIA) database.^[^ ^46^ ^]^ Putative upstream MDK transcription factors were predicted using the hTFtarget,^[^ ^47^ ^]^ HOCOMOCO,^[^ ^48^ ^]^ CIS‐BP,^[^ ^49^ ^]^ and TRANSFAC^[^ ^50^ ^]^ databases. The interaction between SP1 and the MDK promoter was visualized using the Cistrome database.^[^ ^51^ ^]^ The potential binding sites of SP1 within the MDK promoter were predicted and visualized using the JASPAR database.^[^ ^52^ ^]^ Proteomic data from 137 PDAC tissues and 66 adjacent normal tissues were obtained from the CPTAC cohort.^[^ ^53^ ^]^

### 2.22 Statistical Analysis

Statistical analyses were performed using R and GraphPad Prism 9. Kaplan–Meier survival curves were generated using the “survival” R package. All data are presented as mean ± standard deviation (SD) derived from three to four independent experiments. Variances between groups were evaluated using Student's *t*‐test, and associations between groups were evaluated using Pearson's correlation analysis. A significance level of P<0.05 was deemed statistically significant.

## 3 Results

### 3.1 scRNA‐seq Data Analysis and Cell Type Identification

**Figure 1:**
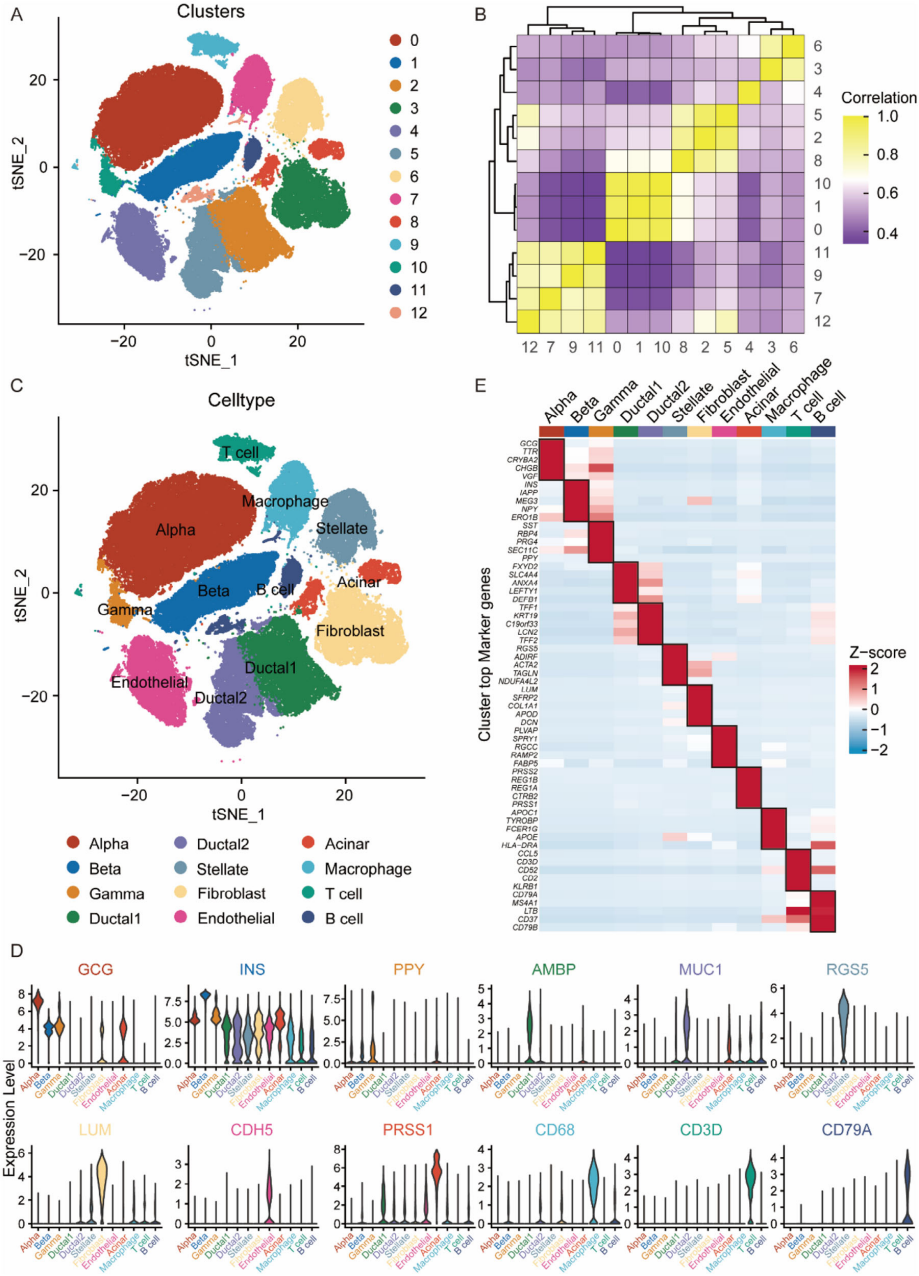
Single‐cell expression atlas of PDAC patients, control samples and human islets. A) t‐SNE plot showing the 12 clusters of 87716 cells from PDAC patients, control samples and human islets. B) The correlations between 12 cell clusters. C) t‐SNE plot of major cell types identified in this study. D) Violin plots visualizing the expression of representative proverbial marker genes in each cell type. E) Heatmap displaying the expression of specific marker genes in each cell type.

To comprehensively explore the functional dynamics of beta cells affected by PDAC cells at single‐cell resolution, we reintegrated and reanalyzed samples from 24 PDAC patients, 11 non‐PDAC control patients, and 14 normal human islets sourced from two distinct studies.^[^ ^41^, ^42^ ^]^ Following initial data quality control, damaged or nonviable cells were excluded, resulting in a cohort of 87716 cells for subsequent analyses (Figure S1A–E, Supporting Information). Harmony successfully mitigated batch effects, with integrated data exhibiting markedly improved sample mixing relative to uncorrected data (Figure S1F, Supporting Information). Subsequently, we employed principal component analysis and t‐SNE to identify 12 distinct cell clusters (**Figure** **1**A). The correlation between gene expression profiles of these different cell clusters is shown in Figure 1B. These cells were characterized into 12 distinct cell types based on well‐established marker genes (Figure 1C), including, for instance, Insulin (INS) for beta cells, and Mucin‐1 (MUC1) for malignant ductal cells, among others (Figure 1D). Specifically, we identified 21172 alpha cells (24.1%), 10644 beta cells (12.1%), 2801 gamma cells (3.2%), 11061 type 1 ductal cells (12.6%), 6759 type 2 ductal cells (7.7%), 5697 stellate cells (6.5%), 9069 fibroblast cells (10.3%), 7699 endothelial cells (8.8%), 2760 acinar cells (3.1%), 4936 macrophage cells (5.6%), 2574 T cells (2.9%), and 2544 B cells (2.9%). Through the utilization of differential gene expression analysis, we identified multiple additional markers for each cell population, such as Maternally expressed gene 3 (MEG3) for beta cells and Trefoil factor 1 (TFF1) for ductal cells (Figure 1E). Consistent with a previous publication, we distinguished two distinct types of ductal cells in this study. Both types of ductal cells expressed canonical ductal markers; however, type 1 cells displayed gene expression patterns resembling normal ductal cells, while type 2 cells exhibited significantly elevated tumor‐promoting markers, suggesting a more malignant phenotype.^[^ ^41^ ^]^

### 3.2 Intercellular Communication Network Analysis for PDAC Tumors and Control Samples

**Figure 2:**
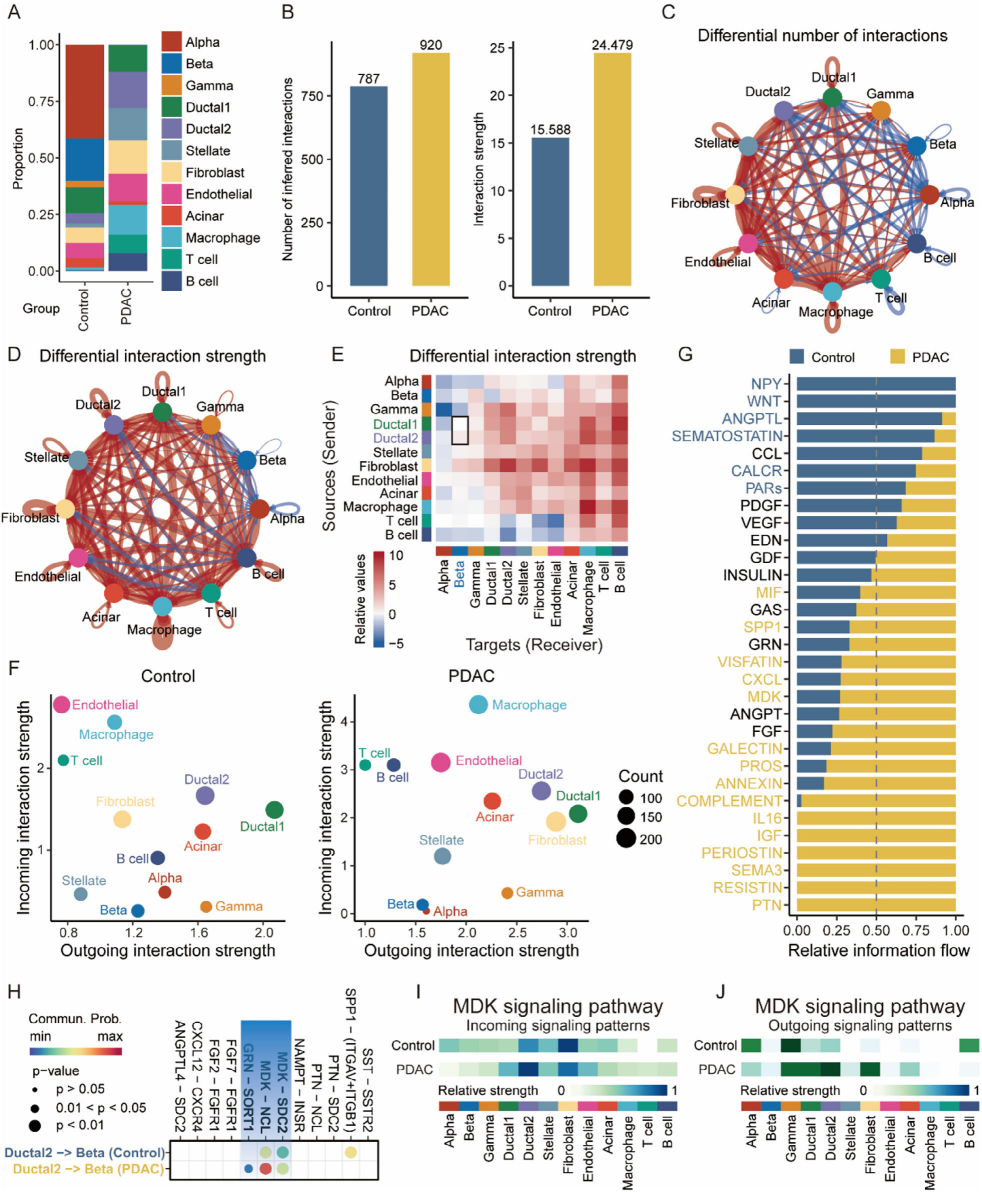
Construction of intercellular communication networks for PDAC tumors and control samples. A) Bar plot showing the proportion of each cell type in PDAC tumors and control samples. B) Bar plot comparing the quantity and strength of cell‐cell interactions in PDAC tumors and control samples. C,D) Circular diagram illustrating the interaction quantity (C) and intensity (D) between PDAC tumors and control samples. E) Heatmap depicting the differential strength of intercellular interactions. F) Scatter chart visualizing the strength of outgoing and incoming interactions. G) All signaling pathways were ranked based on their differences in the relative information flow within the inferred networks between PDAC tumors and control samples. H) Comparison of the significant ligand‐receptor pairs involved in type 2 ductal cell‐beta cell interactions between PDAC tumors and control samples. I,J) Incoming (I) and outgoing (J) patterns of MDK signaling pathway among cell clusters in PDAC tumors and control samples.

To comprehensively discern the discrepancy in cellular composition between PDAC tumors and control samples, we meticulously compared the proportions of each cell subpopulation. As anticipated, a notably augmented representation of type 2 ductal cells and a markedly diminished presence of beta cells were observed in PDAC tumors, in contrast to control samples (**Figure** **2**A; Figure S2A, Supporting Information). The CellChat algorithm requires a minimum threshold of 10 cells per group for robust communication inference.^[^ ^54^ ^]^ With 48 beta cells identified in PDAC samples—exceeding this empirical threshold—the population size was deemed suitable for communication analysis. In addition to cellular composition, differences in cellular communication patterns between PDAC tumors and control samples were investigated. Intercellular interactions were more pervasive and robust in the PDAC samples than in the control samples (Figure 2B). The differential counts and intensities of ligand‐receptor pair interactions among the principal cell types are depicted in Figure 2C,D, respectively. Notably, the interaction potency between type 1 ductal cells and beta cells was not augmented in PDAC samples, whereas that between type 2 ductal cells and beta cells displayed moderate enhancement (Figure 2E). Subsequently, we explored the strength of the incoming and outgoing interactions between cellular populations. In PDAC samples, both the outgoing interaction strength of type 2 ductal cells and the incoming interaction strength of beta cells showed a remarkable increase (Figure 2F). Furthermore, we deduced the potential signaling pathways implicated in intercellular communication based on ligand‐receptor pair interactions (Figure 2G). Signaling pathways that are highly activated in PDAC samples, such as the Pleiotrophin (PTN), Annexin, and Secreted phosphoprotein 1 (SPP1) signaling pathways, have previously been shown to be closely associated with PDAC.^[^ ^55^, ^56^, ^57^ ^]^ Cellular communication between malignant (type 2) ductal cells and beta cells aroused our interest, prompting a detailed exploration of the specific ligand‐receptor interactions between these two cell types. Two putative signaling pathways, MDK and Granulin (GRN), emerged as potential mediators of the interplay between type 2 ductal cells and beta cells in the PDAC samples (Figure 2H). To ascertain whether MDK and GRN play roles in PDAC‐induced paraneoplastic diabetes, we conducted gene expression analysis using the GSE15932 cohort. The results demonstrated that the MDK expression values of MDK in PDAC patients with diabetes were notably higher than those in PDAC patients without diabetes (Figure S2B, Supporting Information). However, GRN expression values of GRN did not significantly upregulated in PDAC patients with diabetes (Figure S2C, Supporting Information), suggesting that only the MDK signaling pathway warrants further investigation. Subsequently, we inferred the incoming and outgoing signaling patterns of the MDK signaling pathway. Beta cells did not receive more MDK signals in PDAC tumors than in control samples (Figure 2I), whereas type 2 ductal cells dispatched more MDK signals to other cells in PDAC tumors than in control samples (Figure 2J). Notably, fibroblasts also appeared to increase MDK outgoing signaling in PDAC (Figure 2J), suggesting potential MDK signals from fibroblasts to beta cells. However, IHC assays revealed MDK protein expression was restricted to tumor cells, with no detectable expression in PDAC stromal components, which comprised fibroblasts (Figure S2D, Supporting Information). This absence precluded fibroblast‐mediated MDK secretion and paracrine signaling to beta cells.

### 3.3 Pseudotime Trajectory Analysis of Ductal Cells in PDAC Samples

**Figure 3:**
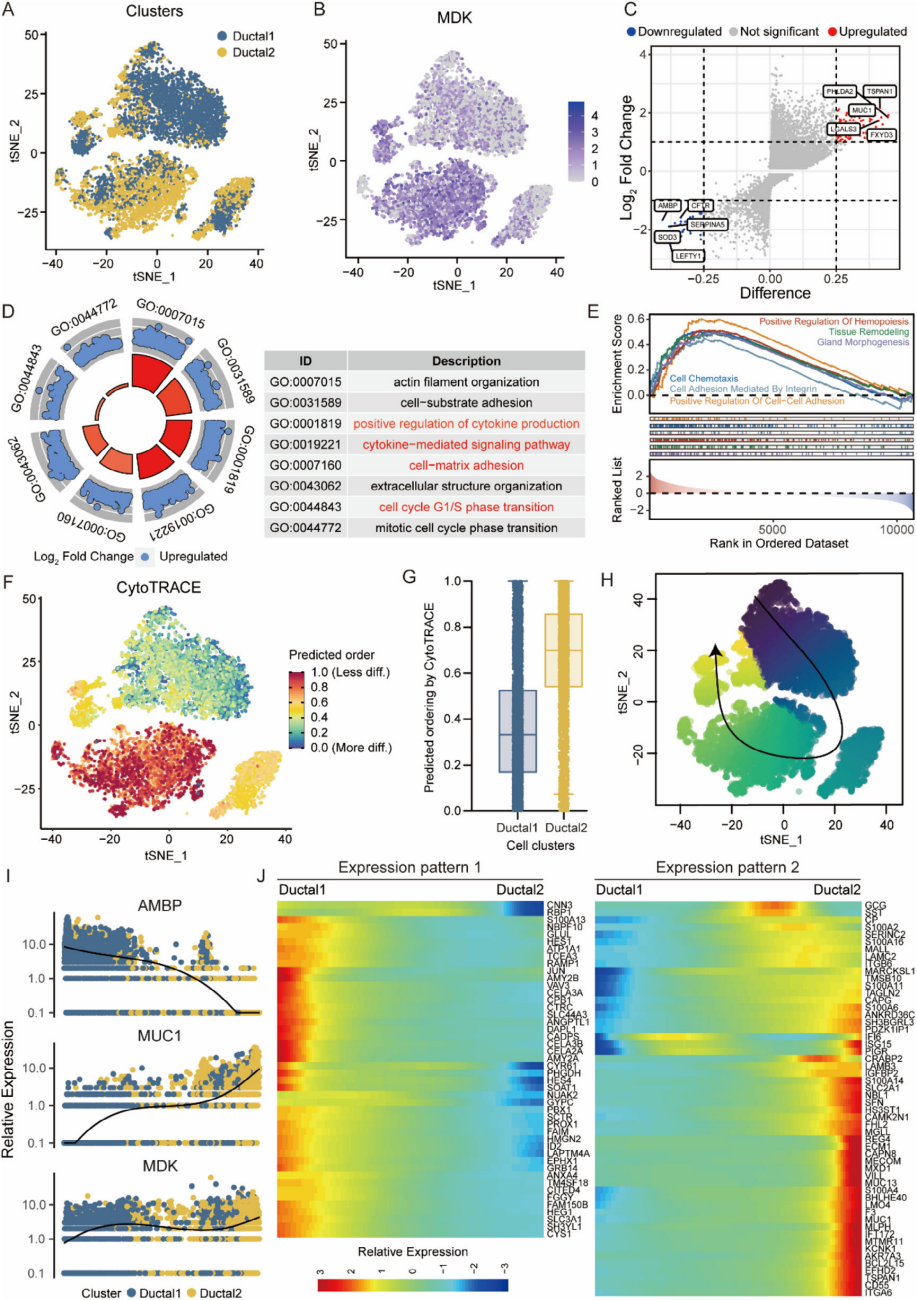
Pseudotime and single‐cell trajectory analyses of ductal cells in PDAC samples. A) t‐SNE plot showing two sub‐clusters of ductal cells. B) t‐SNE plot illustrating the expression of MDK in two ductal cell clusters. C) Volcano plot depicting differentially expressed genes between type 1 and 2 ductal cells. Red dots represent genes expressed at higher levels in type 2 ductal cells while blue dots represent genes with higher expression levels in type 1 ductal cells. D) GO analysis of genes with higher expression levels in type 2 ductal cells. E) GSEA plot displaying the upregulated signaling pathways in type 2 ductal cells. F) t‐SNE plot of CytoTRACE scores showing the distribution of cell stemness from type 1 to type 2 ductal cells. G) Box plot comparing the CytoTRACE values between type 1 and type 2 ductal cells. H) t‐SNE plot depicting the trajectory of ductal cells using Slingshot. I) Dot plots showing dynamic expression of marker genes of type 1 ductal cells (AMBP), type 2 ductal cells (MUC1) and MDK. J) Heatmap showing two expression patterns of differentially expressed genes along the pseudotime.

Considering the plasticity and heterogeneity of ductal cells, we categorized them as type 1 ductal cells exhibiting benign gene expression profiles and type 2 ductal cells displaying malignant gene expression profiles, with predominant MDK expression observed in type 2 ductal cells (**Figure** **3**A,B). The malignancy of type 2 ductal cells was further verified through CNV prediction analysis, which revealed significantly elevated CNV scores in type 2 ductal cells compared with those in type 1 ductal cells (Figure S3, Supporting Information). To elucidate the biological processes and pathways associated with cell malignancy, we identified DEGs between type 1 and type 2 ductal cells, which were subjected to GO analysis and GSEA (Figure 3C). The pathways involved in the regulation of cytokine production, cell adhesion, and cell cycle were altered in type 2 ductal cells (Figure 3D,E). To delineate the dynamic changes in gene expression patterns during malignant progression, we conducted a pseudo‐time trajectory analysis of type 1 and type 2 ductal cells. Type 2 ductal cells demonstrated significantly lower differentiation levels than type 1 ductal cells, indicating the maintenance of elevated stemness within type 2 ductal cells (Figure 3F,G). Figure 3H depicts the speculative developmental relationship between the type 1 and type 2 ductal cells. Throughout the transition from type 1 to type 2 ductal cells, MDK and a type 2 ductal cell marker gene (MUC1) were substantially upregulated, whereas the marker gene of type 1 ductal cells (AMBP) was notably downregulated (Figure 3I). Additionally, two distinct patterns of gene expression were observed during cell state transition (Figure 3J).

### 3.4 Expression of MDK and its Clinical Significance

**Figure 4:**
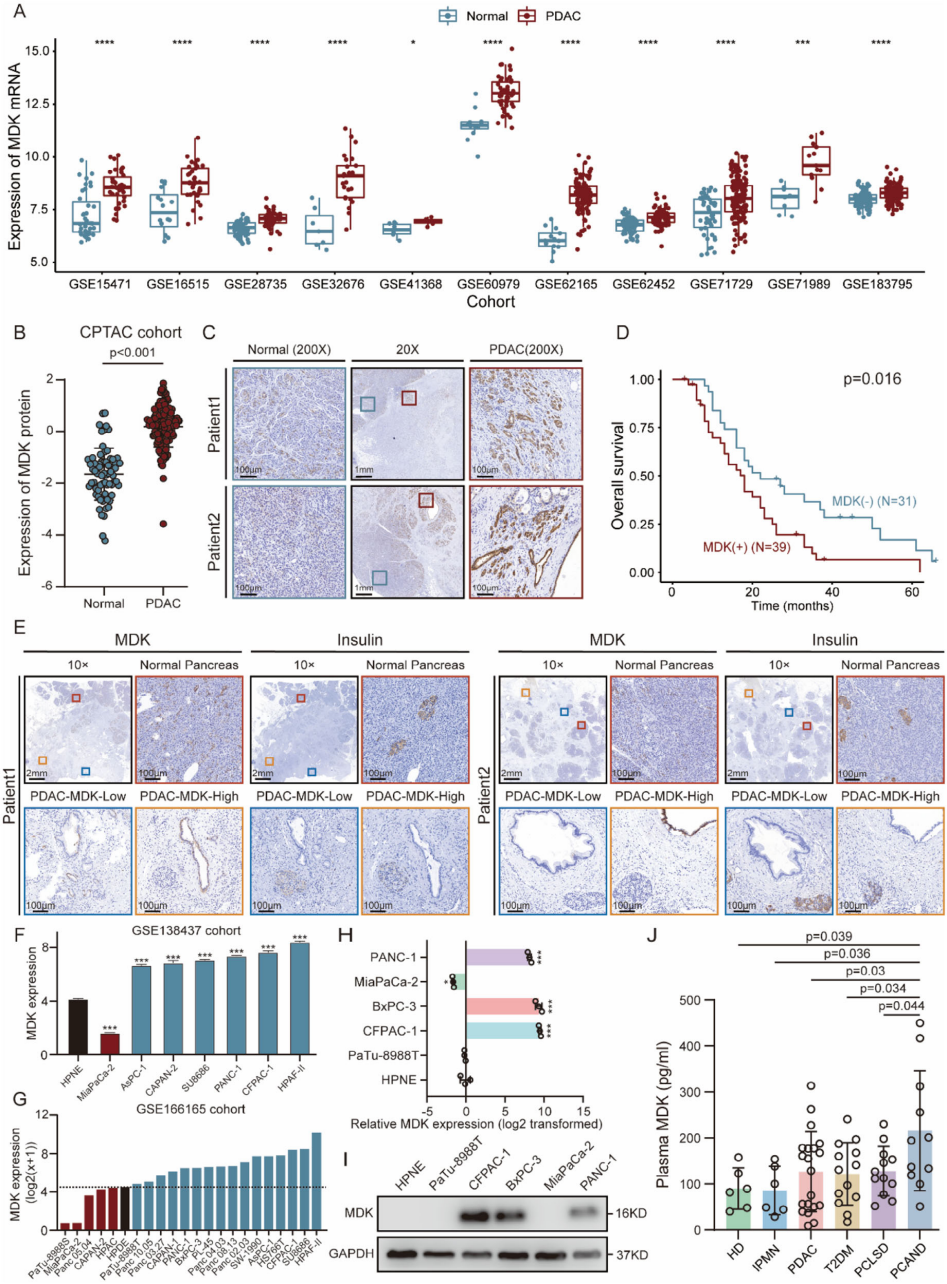
MDK is upregulated in PDAC and has clinical significance. A) MDK mRNA levels in PDAC and adjacent normal tissues from 11 independent cohorts. B) MDK protein levels in PDAC and adjacent normal tissues from CPTAC cohort. C) Representative images of IHC staining of MDK in sections capturing both PDAC and adjacent normal pancreas. D) Kaplan‒Meier curve depicting survival differences between two groups of PDAC patients (n = 70): MDK (+), patients with high MDK expression; MDK (−), patients with low MDK expression. The expression of MDK was determined by IHC score. E) Representative images of IHC staining of MDK and insulin in sections capturing both normal pancreas, PDAC with low MDK expression and PDAC with high MDK expression. F) MDK mRNA levels in normal pancreatic duct cells (HPNE) and PDAC cells from the public GSE138437 cohort. G) MDK mRNA levels in normal pancreatic duct cells (HPDE) and PDAC cells from the public GSE166165 cohort. H,I) MDK mRNA (H) and protein (I) levels detected in PDAC cell lines and HPNE. J) Plasma MDK levels in the peripheral blood samples from healthy donor (HD, N = 6), intraductal papillary mucinous neoplasm (IPMN, N = 6), PDAC without diabetes (PDAC, N = 20), type 2 diabetes mellitus (T2DM, N = 13), PDAC with long‐standing T2DM (PCLSD, N = 12) and pancreatic cancer‐associated new‐onset diabetes (PCAND, N = 11). ^*^ *p* < 0.05; ^**^ *p* < 0.01; ^***^ *p* < 0.001; and ^****^ *p* < 0.0001, means ± SD was shown. Student's *t*‐test analysis was used for comparison between two groups.

In the 11 distinct GEO cohorts (**Figure** **4**A) and the GEPIA database (Figure S4A, Supporting Information), a notable elevation in MDK mRNA levels was observed in PDAC tissues compared to normal tissues. In the CPTAC cohort, discernible upregulation of MDK protein levels was observed in PDAC tissues compared to normal tissues (Figure 4B). Furthermore, IHC staining provided additional validation of MDK overexpression in the PDAC tissues (Figure 4C). Kaplan–Meier survival analysis demonstrated that increased MDK expression correlated with an unfavorable prognosis (Figure 4D). Univariate Cox regression analysis showed that MDK expression served as an independent prognostic factor for overall survival (Figure S4B, Supporting Information). Subgroup survival analyses revealed significantly worse outcomes for PDAC patients with new‐onset diabetes compared to those without diabetes. However, no significant survival difference was observed between PDAC patients with long‐standing diabetes and those without diabetes (Figure S4C, Supporting Information). Notably, our investigation demonstrated a significant inverse correlation between MDK expression in PDAC tissues and insulin expression in adjacent islet tissues (r = −0.3531, p = 0.007), implicating MDK as a potential mediator in pancreatic cancer‐associated beta cell dysfunction (Figure 4E; Figure S4D, Supporting Information). Subsequent examination of MDK expression patterns in cell lines revealed higher MDK levels in PDAC cells than in normal pancreatic duct cells, as evidenced by expression analyses of two independent public datasets (Figure 4F,G). Experimental validation using RT‐qPCR and western blotting confirmed similar MDK expression patterns (Figure 4H,I).

Considering that MDK is a secreted protein detectable in the blood, we explored the potential of plasma MDK levels as a biomarker for PCAND. ELISA results demonstrated that plasma MDK levels were significantly elevated in PCAND patients relative to healthy controls and patients with IPMN, PDAC, T2DM, or PCLSD (Figure 4J). Receiver operating characteristic (ROC) analysis demonstrated moderate diagnostic accuracy for plasma MDK in distinguishing PCAND from T2DM (Area under the curve = 0.72; Figure S4E, Supporting Information). The optimal plasma MDK cutoff was 190.797 pg mL^−1^. Using this threshold (≥190.797 pg mL^−1^), plasma MDK effectively discriminated PCAND from T2DM, with a sensitivity of 54.5% and a specificity of 84.6% (Figure S4E, Supporting Information). Furthermore, plasma proteomics data from the HPA database^[^ ^58^ ^]^ revealed markedly elevated plasma MDK levels in PDAC patients (median: 2.3 NPX, N = 74) compared to both healthy controls (median: −0.1 NPX, N = 832) and T2DM patients (median: 0 NPX, N = 78) (Figure S4F, Supporting Information). These findings supported the utility of MDK as a promising diagnostic biomarker for early PDAC detection.

### 3.5 PDAC Cells Impair Beta Cell Function in an MDK‐Dependent Manner

**Figure 5:**
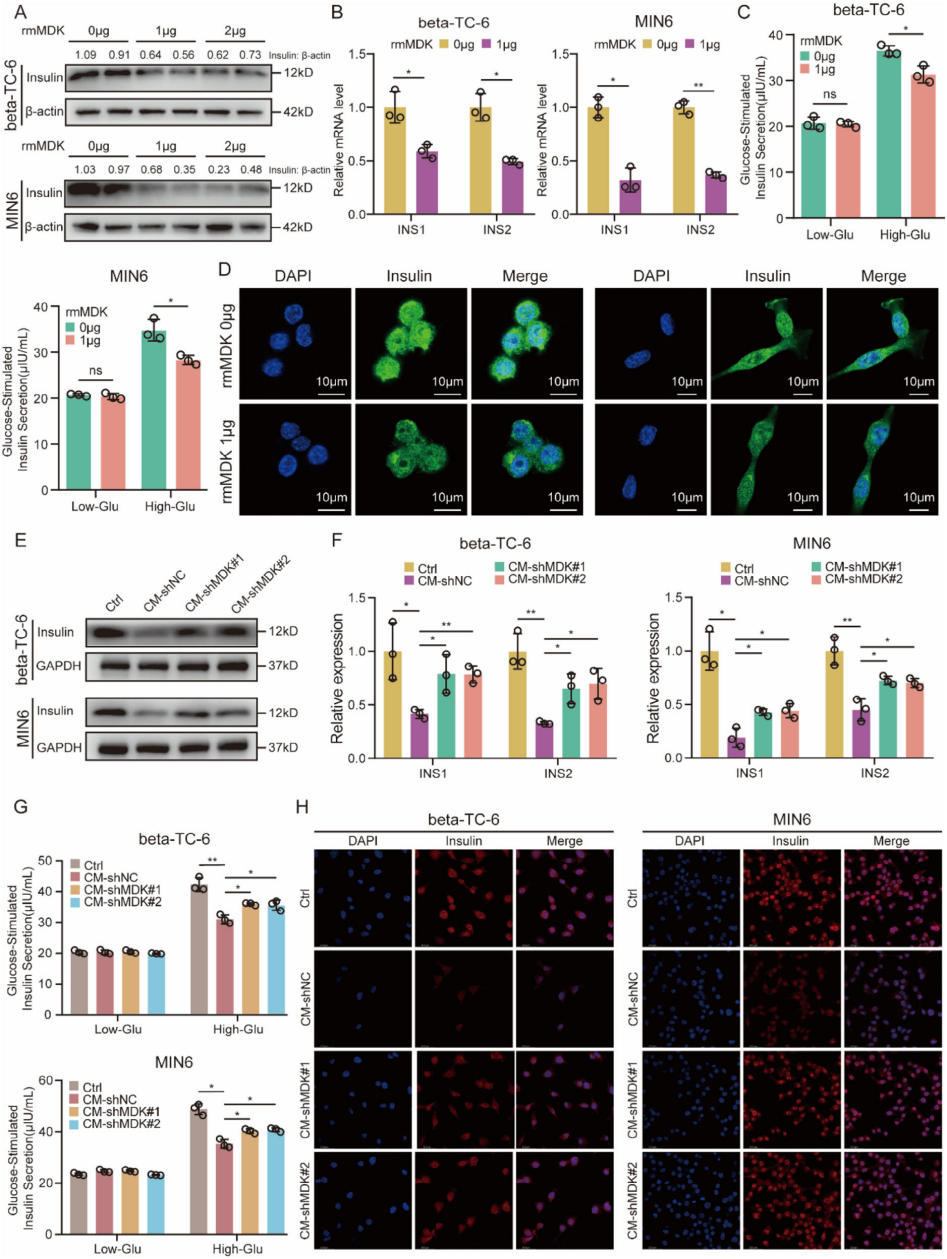
MDK impairs beta cell function in vitro. A) The protein expression levels of insulin in beta cells (beta‐TC‐6 and MIN6) treated with recombinant mouse MDK protein (rmMDK) were detected by western blotting. Beta‐actin was used as the loading control. B) The relative mRNA levels of INS1 and INS2 in beta cells treated with rmMDK were measured by RT‐qPCR. C) Detection of low and high glucose‐stimulated insulin secretion levels in beta cells treated with rmMDK. D) The content of insulin in beta cells treated with rmMDK were determined by immunofluorescence staining. E) Protein expression levels of insulin in beta cells treated with control medium and conditioned mediums from PANC02 cells. GAPDH was used as the loading control. F) The relative mRNA levels of INS1 and INS2 in beta cells treated with different mediums. G) Low and high glucose‐stimulated insulin secretion levels in beta cells treated with indicated mediums. H) The content of insulin in beta cells treated with indicated mediums. ns, not significant; ^*^ *p* < 0.05; and ^**^ *p* < 0.01, means ± SD was shown. Student's t‐test analysis was used for comparison between two groups.

To ascertain the potential involvement of PDAC cell‐derived MDK in paraneoplastic beta cell dysfunction, we used two distinct in vitro culture systems. In one approach, recombinant mouse MDK (rmMDK) protein was directly introduced into the culture medium of recipient beta cells, whereas in the other approach, the normal culture medium was replaced with conditioned medium obtained from PDAC cells. Our functional studies utilized two insulin‐producing and insulin‐secreting beta cell lines, beta‐TC‐6 and MIN6. To assess dose‐dependent effects on insulin levels, beta cells were treated with 1 or 2 µg rmMDK. As shown in **Figure** **5**A, treatment with 1 µg rmMDK effectively reduced insulin protein expression, while no further reduction was observed with 2 µg rmMDK. Consequently, 1 µg rmMDK was standardized for all subsequent beta cell functional assays. Beyond reducing insulin protein levels, 1 µg rmMDK significantly suppressed insulin transcript expression (Figure 5B), impaired glucose‐stimulated insulin secretion (Figure 5C), and decreased insulin content (Figure 5D). Previous investigations have substantiated the inhibitory effect of conditioned medium from PDAC cells on insulin secretion in beta cells.^[^ ^24^, ^35^, ^36^ ^]^ In agreement with these previous findings, our results demonstrated that beta cells co‐cultured in conditioned media from PANC02 cells displayed compromised insulin synthesis and secretion capacity compared to beta cells co‐cultured in control media. Notably, conditioned media from MDK‐knockdown (shMDK#1 and shMDK#2) PANC02 cells significantly mitigated the impairment of beta cell function compared to conditioned media from shNC PANC02 cells (Figure 5E–H). Successful knockdown of MDK in PANC02 cells was validated by RT‐qPCR and western blotting (Figure S5A,B, Supporting Information). Collectively, the aforementioned in vitro functional assays strongly indicated that PDAC‐secreted MDK serves as a trigger for paraneoplastic beta cell dysfunction.

**Figure 6:**
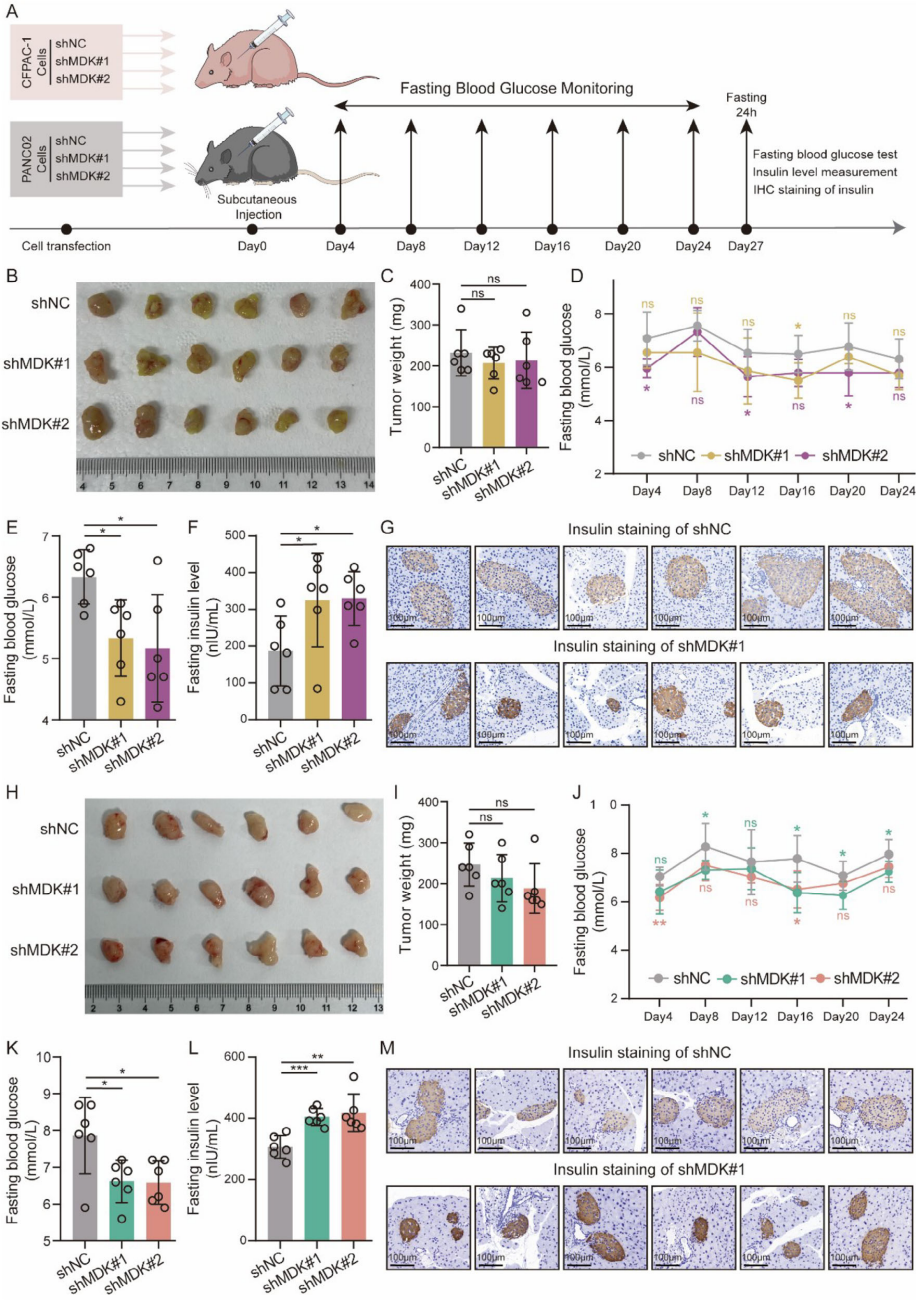
MDK impairs beta cell function in vivo. A) Workflow of in vivo animal studies. B,H) Representative images of subcutaneous tumors formed by the indicated cells in nude mice (B) and C57BL/6 mice (H). C,I) Weights of excised tumors in nude mice (C) and C57BL/6 mice (I) are measured. D,J) Fasting blood glucose (fasting 6 h before blood sampling) monitoring was performed every 4 days after cell injection in nude mice (D) and C57BL/6 mice (J). E,K) Detection of fasting blood glucose (fasting 24 h before blood sampling) in nude mice (E) and C57BL/6 mice (K) before sacrifice. F,L) Measurement of fasting insulin levels (fasting 24 h before blood sampling) in nude mice (F) and C57BL/6 mice (L) before sacrifice. G,M) Representative images of IHC staining of insulin in pancreases from nude mice (G) and C57BL/6 mice (M). ns, not significant; ^*^ *p* < 0.05; ^**^ *p* < 0.01; and ^***^ *p* < 0.001, means ± SD was shown. Student's *t*‐test analysis was used for comparison between two groups.

To substantiate the potential role of MDK in perturbing glucose homeostasis, we performed in vivo functional assays using two established murine PCAND models: subcutaneous and orthotopic xenografts.^[^ ^59^ ^]^ In the subcutaneous model, human CFPAC‐1 PDAC cells were injected into nude mice, whereas mouse PANC02 PDAC cells were injected into C57BL/6 mice (**Figure** **6**A). The efficacy of MDK knockdown in CFPAC‐1 cells was confirmed using RT‐qPCR and western blotting (Figure S6A,B, Supporting Information). Notably, in both nude and C57BL/6 mice, no significant differences were observed in the volume and weight of tumors formed by MDK‐silenced cells compared with those formed by control cells (Figure 6B,C,H,I). In contrast to the mice injected with control cells, those injected with MDK‐silenced cells exhibited moderately reduced fasting blood glucose levels at various time points post‐injection (Figure 6D,J). Following a 24‐h fasting period before sample collection, mice injected with MDK‐silenced cells displayed diminished fasting blood glucose levels (Figure 6E,K), elevated fasting insulin levels (Figure 6F,L), and increased IHC staining intensity of insulin (Figure 6G,M). In the orthotopic model, luciferase‐expressing CFPAC‐1 cells were injected; the resulting tumors formed by MDK‐depleted cells had volumes comparable to those formed by control cells (Figure S6F,G, Supporting Information). Consistent with subcutaneous findings, mice bearing orthotopic shMDK tumors exhibited decreased fasting blood glucose levels and increased fasting insulin levels compared to shNC controls, further supporting MDK's detrimental role in islet function (Figure S6H–J, Supporting Information). Western blotting confirmed MDK knockdown efficiency in CFPAC‐1 subcutaneous tumors, PANC02 subcutaneous tumors, and CFPAC‐1 orthotopic tumors (Figure S6C–E, Supporting Information). These in vivo functional assays jointly indicated that PDAC cells induce beta cell dysfunction in an MDK‐dependent manner.

### 3.6 MDK‐SDC4 Interaction Damages Beta Cell Function by Inhibiting Ras/Raf/MEK/ERK Signaling Pathway

**Figure 7:**
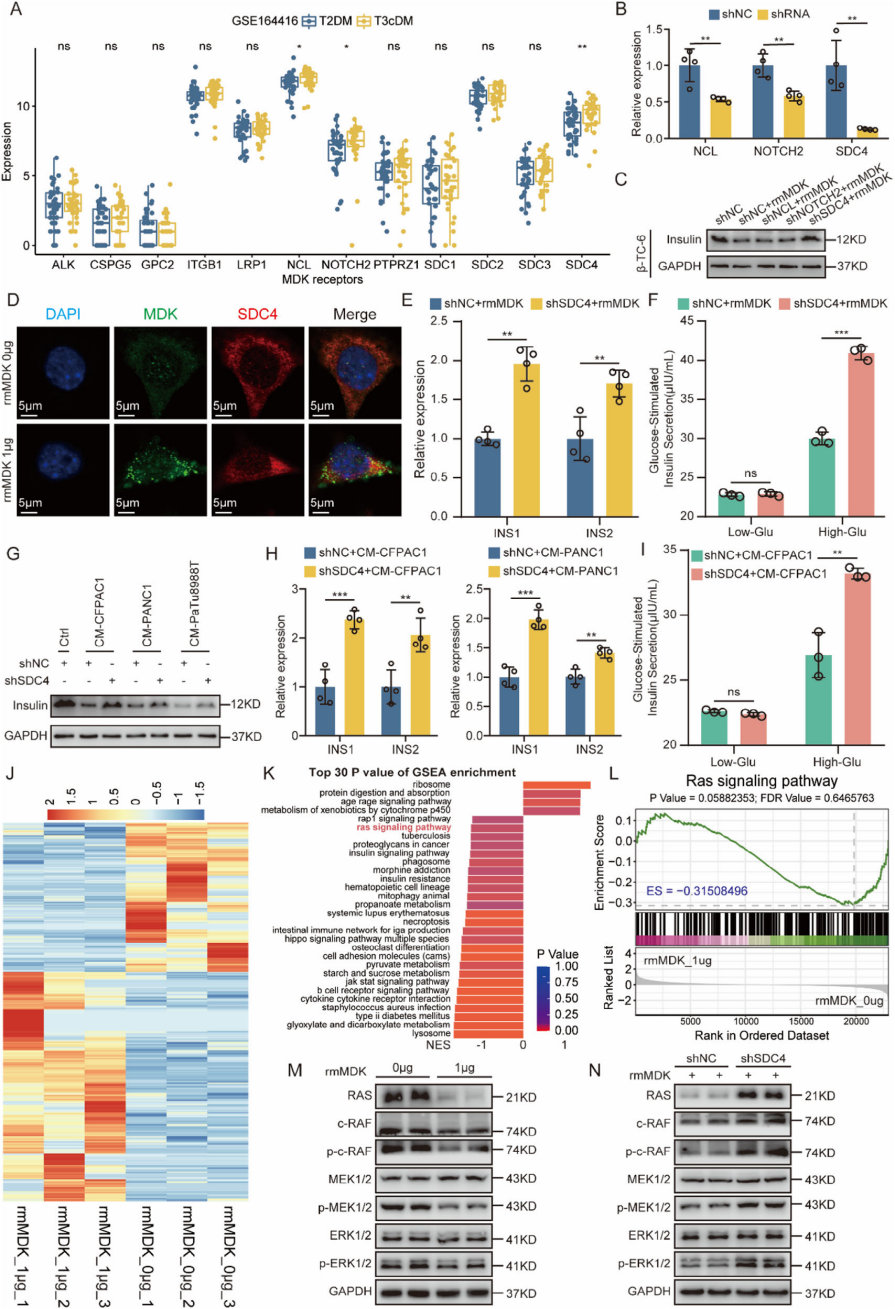
MDK‐SDC4 interaction damages beta cell function by inhibiting Ras signaling pathway. A) Expression of potential MDK receptors in human islets from T2DM and T3cDM patients. B) Knockdown efficiency of NCL, NOTCH2 and SDC4 in beta‐TC‐6 cells was determined by RT‐qPCR. C) The protein levels of insulin in beta‐TC‐6 cells. D) The interaction between MDK and SDC4 was detected by the immunofluorescence assay in beta‐TC‐6 cells. E) The relative mRNA levels of INS1 and INS2 in the indicated beta cells treated with rmMDK. F) Low and high glucose‐stimulated insulin secretion levels in the indicated beta cells treated with rmMDK. G) The protein levels of insulin in the indicated cells treated with control medium and conditioned mediums from PDAC cells. H) The relative mRNA levels of INS1 and INS2 in the indicated cells treated with conditioned mediums from CFPAC‐1 cells (left panel) and PANC1 cells (right panel). I) Low and high glucose‐stimulated insulin secretion levels in the indicated beta cells treated with conditioned mediums from CFPAC‐1 cells. J) Heatmap showing differentially expressed genes between rmMDK‐treated and control beta‐TC‐6 cells. K) Bar plot displaying top 30 enriched signaling pathways in the GSEA analysis. L) GSEA plot of Ras signaling pathway. M) Western blotting analysis of the Ras/Raf/MEK/ERK signaling pathway in beta‐TC‐6 cells treated with rmMDK. N) Western blotting analysis of the Ras/Raf/MEK/ERK signaling pathway in the indicated beta‐TC‐6 cells treated with rmMDK. ns, not significant; ^*^ *p* < 0.05; ^**^ *p* < 0.01; and ^***^ *p* < 0.001, means ± SD was shown. Student's *t*‐test analysis was used for comparison between two groups.

To elucidate the potential receptors mediating the effect of MDK on beta cells, an exhaustive review of the literature revealed 12 experimentally validated MDK receptors (Table S6, Supporting Information). Given the upregulation of MDK in patients with PCAND (Figure 4J), we hypothesized that MDK receptors might manifest augmented expression in the islets of patients with PCAND. Leveraging the GSE164416 cohort, encompassing human islet gene expression data from 39 patients with T2DM and 35 patients with T3cDM, offered an opportunity to narrow down the potential receptors. Notably, among the 35 T3cDM donors, 26 were PCAND samples, while the remaining nine non‐PCAND samples could not be excluded owing to insufficient clinical information. While acknowledging the potential for bias introduced by utilizing this cohort for expression analysis, we deemed it acceptable given the high percentage of PCAND, accounting for 74.29% of the dataset. The expression levels of three potential receptors—namely, NCL, NOTCH2, and SDC4—were markedly elevated in the islets of patients with T3cDM (**Figure** **7**A). To substantiate these bioinformatic findings, we employed specific shRNAs to silence these three receptors in beta‐TC‐6 cells (Figure 7B). The results revealed that knockdown of SDC4 alone ameliorated the deleterious effect of rmMDK on beta‐TC‐6 cells (Figure 7C), thereby prompting the selection of SDC4 for further investigation. Previous studies show SDC4 is predominantly expressed in pancreatic beta cells, with no detectable expression in alpha or delta cells of human and mouse islets. While absent in mouse pancreatic gamma cells, SDC4 presents in a minority of human gamma cell.^[^ ^60^, ^61^ ^]^ scRNA‐seq analysis confirmed SDC4 abundance in beta cells, negligible expression in gamma cells, and absence in alpha cells (Figure S7A, Supporting Information). IF staining of PDAC tissues revealed significant SDC4/insulin co‐localization (Pearson's r = 0.945; Figure S7B, Supporting Information). Furthermore, rmMDK treatment did not alter glucagon mRNA or protein levels in alpha TC‐1 cells (Figure S7C,D, Supporting Information). These findings confirmed beta cell‐specific SDC4 expression, suggesting exclusive MDK‐SDC4 interactions in beta cells. The interaction between MDK and SDC4 was subsequently confirmed by IF (Figure 7D) and co‐IP assays (Figure S7E, Supporting Information). Knockdown of SDC4 significantly attenuated the suppressive effects of rmMDK on insulin expression and the GSIS response, suggesting that MDK‐mediated beta cell dysfunction was dependent on SDC4 (Figure 7E,F). Notably, the inhibitory effects of conditioned medium derived from wild‐type PDAC cells on insulin protein (Figure 7G), insulin mRNA (Figure 7H), and insulin secretion (Figure 7I; Figure S7F, Supporting Information) were partially alleviated by SDC4 knockdown.

In order to preliminarily unravel the underlying mechanism by which MDK‐SDC4 interactions exacerbated beta cell dysfunction, we carried out RNA‐seq analysis on beta‐TC‐6 cells, subjecting them to treatment with or without rmMDK. Notably, principal component analysis distinguished the rmMDK‐treated cells from their control counterparts (Figure S8A, Supporting Information). Examination of DEGs revealed 226 upregulated and 147 downregulated genes in rmMDK‐treated cells (Figure 7J; Figure S8B, Supporting Information). Subsequent GSEA revealed the downregulation of multiple signaling pathways following rmMDK treatment (Figure 7K). Notably, among these pathways was the Ras signaling pathway (Figure 7L), which captured our attention because of the close association between the ERK1/2 cascade and rmMDK treatment, as revealed by GO analysis of DEGs (Figure S8C, Supporting Information). Pharmacological inhibition of Ras signaling using two distinct inhibitors (MCP110 and Pan‐RAS‐IN‐1) significantly suppressed both insulin gene expression and secretion, confirming its essential role in maintaining basal beta cell function (Figure S8D–F, Supporting Information). As shown in Figure 7M, rmMDK treatment markedly suppressed Ras expression and the phosphorylation of c‐Raf, MEK1/2, and ERK1/2, but these inhibitory effects were fully reversed by co‐administration of the Ras agonist ML‐098 (Figure S8G–I, Supporting Information). Additionally, SDC4‐silenced beta cells, upon exposure to rmMDK, exhibited elevated Ras expression and augmented phosphorylation of c‐Raf, MEK1/2, and ERK1/2 compared to control beta cells (Figure 7N). Ras signaling promotes Pdx1 and MafA expression in beta cells,^[^ ^62^ ^]^ whereas rmMDK treatment significantly inhibited expression of both Pdx1 and MafA (Figure S8J,K, Supporting Information). These findings collectively highlighted the damaging effect of MDK‐SDC4 interactions on beta cells through the Ras/Raf/MEK/ERK signaling cascade.

To elucidate the molecular mechanisms underlying the Ras signaling‐suppressive effect mediated by the MDK‐SDC4 interaction, we focused on DEGs identified by RNA‐seq comparing rmMDK‐treated and untreated groups. Among the top‐ranked DEGs, Caveolin‐1 (Cav1) was prioritized for further investigation. Cav1 is known to suppress basal insulin production and secretion by inhibiting the Ras/Raf/MEK/ERK signaling pathway in pancreatic beta cells.^[^ ^63^, ^64^, ^65^ ^]^ Western blotting and RT‐qPCR revealed that rmMDK treatment significantly upregulated Cav1 expression in beta cells (Figure S8L,M, Supporting Information).To determine whether Cav1 depletion could reverse the rmMDK‐mediated inhibition of Ras signaling, we silenced Cav1 using shRNA. The results demonstrated that Cav1 depletion fully restored Ras signaling pathway activity inhibited by rmMDK treatment (Figure S8N, Supporting Information). Collectively, these findings reinforced the interaction between MDK and SDC4 and revealed that the MDK‐SDC4 axis modulated Ras signaling pathway activity through the regulation of Cav1.

### 3.7 SP1 Transcriptionally Activates MDK in PDAC Cells

**Figure 8:**
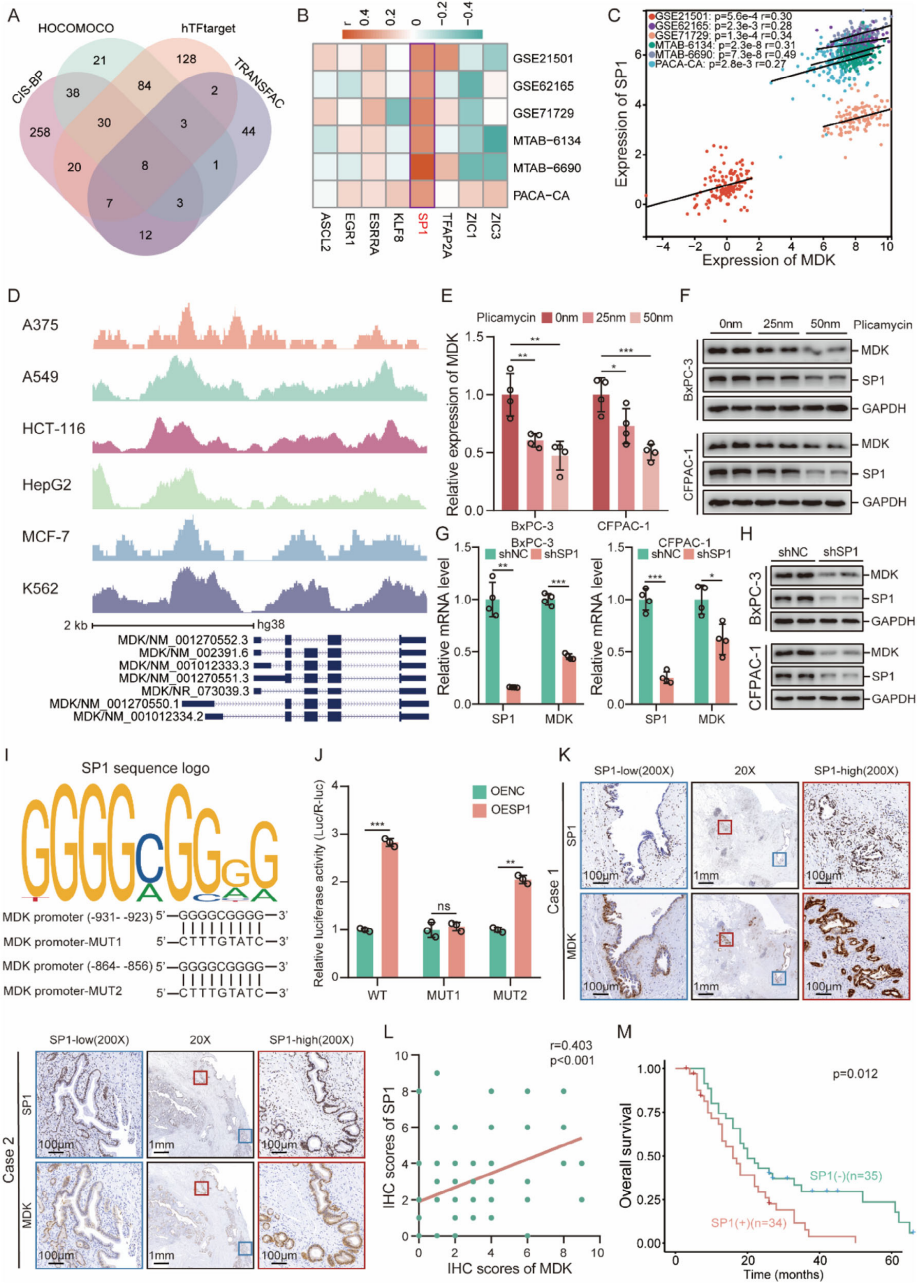
MDK is a downstream target of SP1. A) Venn diagram showing screening strategy for TFs of MDK. B,C) Heatmap (B) and scatter plot (C) visualizing the positive correlations between MDK expression and SP1 expression in six public PDAC cohorts. D) The distribution of SP1 peaks across MDK promoter acquired from freely accessible chromatin immunoprecipitation‐sequencing data. E) RT‐qPCR analysis of MDK mRNA levels in BxPC‐3 and CFPAC‐1 cells treated with Plicamycin. F) Western blotting analysis of MDK and SP1 protein levels in BxPC‐3 and CFPAC‐1 cells treated with Plicamycin. G) RT‐qPCR analysis of MDK and SP1 mRNA levels in BxPC‐3 and CFPAC‐1 cells with SP1 depletion. H) Western blotting analysis of MDK and SP1 protein levels in BxPC‐3 and CFPAC‐1 cells with SP1 depletion. I) The sequence logo graph illustrated the canonical binding sites of SP1 predicted by JASPAR. J) Relative luciferase activities in HEK‐293T cells transfected with the indicated plasmids. K) Representative IHC staining images of SP1 and MDK. L) Correlation between MDK levels and SP1 levels in our PDAC cohort. M) Kaplan‒Meier curve showing the overall survival of PDAC patients with low (‐)/high (+) SP1 expression. ^*^ *p* < 0.05; ^**^ *p* < 0.01; and ^***^ *p* < 0.001, means ± SD was shown. Student's *t*‐test analysis was used for comparison between two groups.

To elucidate the mechanisms governing MDK overexpression in PDAC, we explored potential transcriptional regulators of MDK using publicly available databases (Figure S9A, Supporting Information). Using the MDK promoter sequence, we predicted potential transcription factors (TFs) by querying four independent databases: hTFtarget, CISBP, HOCOMOCO, and TRANSFAC. Venn diagram analysis identified eight TFs common to all four databases: ASCL2, EGR1, ESRRA, KLF8, SP1, TFAP2A, ZIC1, and ZIC3 (**Figure** **8**A). Among these candidate TFs, only SP1 expression showed a significant positive correlation with MDK expression in all six public PDAC datasets (Figure 8B,C). The putative binding region of SP1 protein to the MDK promoter is shown in Figure 8D. To validate these bioinformatics‐derived insights, we used specific inhibitors or targeted shRNAs to suppress SP1 expression in PDAC cells. The results revealed that the SP1 inhibitor plicamycin dose‐dependently suppressed both SP1 and MDK expression (Figure 8E,F; Figure S9B, Supporting Information). Furthermore, shRNA‐mediated SP1 depletion in PDAC cells resulted in a notable reduction in MDK mRNA and protein levels (Figure 8G,H). Conversely, SP1 overexpression markedly increased MDK mRNA and protein expression (Figure S9C,D, Supporting Information). Considering the distribution of SP1 peaks across the MDK promoter sequence, we assumed potential SP1 binding sites of ≈−1000 bp (Figure 8D). Consequently, sequences from −931 to −923 bp (MUT1) and −864 to −856 bp (MUT2), identified by the JASPAR database as typical binding sites for SP1, were selected for further analysis (Figure 8I). Subsequent luciferase reporter assays further validated SP1's binding to the −931 to −923 bp region of the MDK promoter, thereby activating its expression (Figure 8J). Additionally, a positive correlation between SP1 and MDK proteins was observed in PDAC tissues (Figure 8K,L). According to the GEPIA database, SP1 was significantly upregulated in pancreatic adenocarcinomas compared to that in normal pancreases (Figure S9E, Supporting Information). Importantly, elevated MDK expression was associated with a poor prognosis in patients with PDAC (Figure 8M). Collectively, these findings demonstrated that SP1 is a direct upstream regulator of MDK in PDAC.

## 4 Discussion

Early detection of resectable PDAC is the only method to achieve a cure for this disease. Clinical guidelines recommend PDAC screening for individuals deemed to be at high risk, particularly those presenting with new‐onset diabetes.^[^ ^66^, ^67^, ^68^ ^]^ However, given the higher prevalence of T2DM in the new‐onset diabetes population and the similarity in clinical manifestations between T2DM and PCAND, it is imperative for PDAC research to prioritize the discovery of novel biomarkers that exhibit optimal specificity and sensitivity in discriminating PCAND from T2DM. PCAND is viewed as an outward manifestation of the molecular interplay between beta and tumor cells. Therefore, investigating the driver genes and signaling pathways involved in these cellular interactions presents a valuable opportunity to distinguish PCAND from the more prevalent T2DM.

scRNA‐seq presents an unparalleled opportunity for a comprehensive understanding of cellular diversity, intercellular communication, and evolutionary dynamics of cancer. Within the realm of PDAC, scRNA‐seq has substantially advanced our understanding of cellular constituents and intercellular interactions, including but not limited to the interplay between tumor cells and macrophages as well as interactions involving cancer‐associated fibroblasts and tumor cells.^[^ ^69^, ^70^, ^71^ ^]^ However, studies utilizing scRNA‐seq to specifically decipher the interactions between tumor and beta cells remain limited. In this study, we analyzed publicly available scRNA‐seq data and constructed cell communication networks for PDAC tumor and control samples. Our findings identified MDK as a potential mediator of molecular interactions between malignant ductal cells and beta cells in PDAC samples. Subsequent in vitro and in vivo functional assays collectively affirmed the indispensable role of MDK in tumor cell‐induced beta cell dysfunction. Of particular significance, our observations revealed that, in several tissue sections encompassing both PDAC tumors and adjacent islets, heightened MDK expression in tumors coincided with reduced insulin expression in para‐tumor islets. Furthermore, elevated plasma MDK levels were detected in PCAND samples compared with those in T2DM samples. In summary, our results highlight the close association between MDK and PCAND at the cellular, tissue, and blood levels.

Analysis of cell‐cell communication revealed a discrepancy: type 2 ductal cells exhibited significantly enhanced MDK signaling output to other cell types in PDAC samples compared to controls (Figure 2J), whereas beta cells did not show a corresponding increase in MDK signaling reception in PDAC (Figure 2I). Critically, CellChat computes pathway‐level communication probabilities by aggregating the inferred probabilities for all known ligand‐receptor pairs within that pathway.^[^ ^44^, ^54^ ^]^ For the MDK pathway, this encompasses interactions with its multiple cognate receptors (e.g., NCL, LRP1, SDC1, SDC2, and SDC4). Therefore, the “incoming MDK signaling” depicted in Figure 2I represents the total inferred probability of MDK pathway activation via any of its potential receptors, not solely the MDK‐SDC4 axis highlighted in our study. Consequently, the relatively elevated total MDK signal reception observed in control beta cells relative to PDAC beta cells may reflect potentially higher expression levels of one or more alternative MDK receptors in the control samples, leading CellChat to infer a greater overall probability of MDK signaling engagement.

MDK, a heparin‐binding growth factor cytokine, has been identified as a pivotal contributor to cancer progression and is a promising biomarker for cancer diagnosis and prognosis.^[^ ^72^ ^]^ However, investigations on the relationship between MDK and T2DM remain limited, suggesting that MDK may serve as a diabetogenic factor specific to PCAND. Previous studies have demonstrated that MDK overexpression indicates poor prognosis and facilitates perineural invasion, chemoresistance, proliferation, and migration in PDAC.^[^ ^73^, ^74^, ^75^ ^]^ Consistent with these findings, our study also observed the upregulation of MDK and its prognostic significance (Figure 4A–D); however, the absence of a promotive effect of MDK on tumor growth (Figure 6B,H) suggests potential disparities attributable to substantial biological distinctions between in vivo and in vitro cell culture environments, including factors such as the influence of growth factors and modulation of the immune system.

Our in vivo PCAND results revealed significantly greater fasting blood glucose differences between shMDK and shNC groups after 24‐h versus 6‐h fasts, suggesting prolonged fasting better uncovers intergroup glycemic disparities. Although guidelines propose that 6‐h fasts minimize food ingestion‐induced variability in basal glycemia,^[^ ^76^ ^]^ several studies report no significant blood glucose alterations at 6 h, with significant reductions necessitating ≥12‐h fasts.^[^ ^77^ ^]^ Moreover, shorter fasts may be less effective due to residual food consumption: 18–36% of food is spilled into cage bottoms, accounting for up to 40% of consumption, potentially confounding glucose measurements.^[^ ^78^ ^]^ A 24‐h fast ensures more complete elimination of dietary confounders, likely explaining the enhanced intergroup differences observed. Notably, Ayala et al. demonstrated that 18‐h fasts significantly increase insulin sensitivity versus 5‐h fasts, amplifying intergroup glycemic differences.^[^ ^79^ ^]^ This effect of fasting on insulin action is a well‐documented phenomenon observed in humans and mouse models,^[^ ^80^ ^]^ and may further contribute to the heightened glycemic differences detected under prolonged fasting conditions.

SDC4, the gene encoding the MDK receptor on the surface of beta cell membranes, was identified through a combination of bioinformatic analysis and experimental screening. Initially, we identified 12 experimentally validated MDK receptors (Table S6, Supporting Information) based on the current literature and compared their expression in human islets from 39 patients with T2DM and 35 patients with T3cDM, of which 26 were PCAND. Subsequently, three potential receptors (NCL, NOTCH2, and SDC4) were initially identified because of their higher expression in T3cDM; however, the results might have been influenced by the presence of nine non‐PCAND samples within the T3cDM group, which could have introduced bias. Fortunately, we found that knockdown of SDC4 significantly mitigated the deleterious effects of rmMDK and conditioned media from PDAC cells on beta cells. SDC4, a well‐established MDK receptor, is closely associated with the development of various diseases, including colorectal and ovarian cancer.^[^ ^81^, ^82^, ^83^ ^]^ In line with previous studies,^[^ ^60^, ^61^ ^]^ we detected the expression of SDC4 protein in beta cells. Previous studies have indicated that SDC4 knockdown diminishes the insulin secretory response in MIN6 cells.^[^ ^84^ ^]^ In this study, the MDK‐SDC4 axis impaired the insulin secretory response of beta cells, suggesting that the regulatory effects of SDC4 on insulin secretion may be contingent upon external stimuli.

The RNA‐seq results demonstrated that treatment with rmMDK led to notable inhibition of the Ras signaling pathway. Subsequent western blot analyses confirmed that rmMDK treatment reduced the expression of Ras and the phosphorylation levels of c‐Raf, MEK1/2, and ERK1/2. The Ras/Raf/MEK/ERK signaling cascade orchestrates various cellular processes, including cell proliferation and survival.^[^ ^85^ ^]^ Notably, in pancreatic beta cells, the Ras/Raf/MEK/ERK signaling pathway plays a critical role in the transcription of insulin genes and secretion of insulin.^[^ ^62^ ^]^ Therefore, suppression of the Ras signaling pathway following rmMDK treatment may elucidate, in part, the detrimental impact of MDK‐SDC4 interactions on beta cells. In the pathogenesis of osteoarthritis, SDC4 is upregulated by activation of the Ras signaling pathway.^[^ ^86^ ^]^ Nonetheless, limited research exists regarding the modulatory role of SDC4 in the Ras signaling pathway, particularly within beta cells. Our findings revealed that the attenuation of SDC4 in beta cells ameliorated the inhibition of the Ras signaling pathway induced by rmMDK. This discovery provides new insights into the regulatory mechanisms of the Ras signaling pathway.

Furthermore, we identified SP1 as an upstream regulator of MDK expression. Our analysis revealed positive correlations between SP1 and MDK in both the public datasets and our in‐house cohort. This positive regulatory relationship was further established in PDAC cells by manipulating SP1 expression using knockdown and overexpression systems. Notably, we successfully identified the binding sites of SP1 on the MDK promoter. SP1, a well‐established oncogenic transcription factor in PDAC, stimulates cell proliferation and migration, and confers gemcitabine resistance.^[^ ^87^, ^88^, ^89^ ^]^ While extensive literature confirms that both SP1 and MDK independently drive PDAC metastasis and chemoresistance, the potential MDK‐dependency of SP1‐mediated oncogenic regulation has not been experimentally established. Future studies should employ in vitro and in vivo rescue experiments to characterize the functional relationship within the SP1‐MDK axis and its contribution to PDAC malignancy. Additionally, in gliomas, SP1 activates MDK transcription via promoter binding.^[^ ^90^ ^]^

PCAND typically manifests 2–3 years prior to the diagnosis of PDAC, most likely during the progression from high‐grade pancreatic intraepithelial neoplasia (PanIN) to invasive carcinoma.^[^ ^91^ ^]^ PanIN lesions have been demonstrated to correlate with an elevated incidence of diabetes. Specifically, diabetes was identified in 11% of patients with PanIN lesions, compared to 2% in patients without such lesions.^[^ ^92^ ^]^ Furthermore, Matsuda et al. reported that high‐grade PanIN‐3 lesions were significantly more prevalent in patients with diabetes, whereas low‐grade PanIN‐1 and PanIN‐2 lesions demonstrated no significant association with diabetes.^[^ ^93^ ^]^ Collectively, these clinical observations indicate that while low‐grade PanINs may not contribute to new‐onset diabetes, high‐grade PanIN‐3 lesions are likely implicated in islet cell damage, subsequently leading to glycemic dysregulation. Building upon these clinical findings, and considering that MDK expression is negligibly detectable in low‐grade PanIN lesions, initiates at the PanIN‐3 stage, and progressively increases through to PDAC (Figure S4G, Supporting Information), we propose that interaction of the MDK‐SDC4 axis likely occurs during the progression from PanIN‐3 to PDAC. This axis may provide a critical time window for the early detection of PDAC.

Evidence increasingly distinguishes the prognostic impact of diabetes duration in PDAC. PCAND, frequently a paraneoplastic manifestation, is consistently associated with poorer outcomes, including shorter overall survival and higher recurrence risk after resection, often as an independent predictor.^[^ ^94^ ^]^ Our findings corroborate this, demonstrating a significant association between PCAND and poor prognosis. However, the association between long‐standing diabetes and PDAC prognosis remains ambiguous. While some studies suggest diminished survival, others find no significant effect.^[^ ^95^ ^]^ This inconsistency likely stems from heterogeneity in long‐standing diabetes populations and the strong confounding influence of factors like tumor size.^[^ ^96^ ^]^ Furthermore, our study identified MDK and SP1 protein expression as significant indicators of poor PDAC prognosis. Notably, patients exhibiting concomitant high expression of both MDK and SP1 demonstrated the worst outcomes (Figure S9F, Supporting Information). While the prognostic value of SP1 in PDAC is well‐established, evidence for MDK is less extensive. Our work provides the first large‐scale validation of the association between MDK protein levels and survival, complementing the prior mRNA‐level findings by Yao et al.^[^ ^74^ ^]^ SDC4, a documented oncogene in PDAC, exhibits ubiquitous expression within the tumor microenvironment (cancer cells, stellate cells, fibroblasts, and islets), and its overexpression in both cancer and stromal cells correlates with reduced survival.^[^ ^97^, ^98^ ^]^ This widespread expression complicates survival analysis based on islet cell‐specific SDC4 due to potential confounding by expression in other cell types. Although we performed IHC staining of insulin to identify islets, the limited sample size precluded meaningful survival analysis based on MDK/insulin co‐expression status. Future studies with expanded cohorts are warranted to investigate this.

The emerging consensus in PDAC transcriptomics recognizes two principal subtypes with clinical relevance: classical and basal. These subtypes demonstrate robust prognostic stratification, with basal tumors associating with inferior survival and exhibiting reduced response to fluorouracil‐based therapies.^[^ ^99^ ^]^ However, the relationship between these transcriptomic subtypes and new‐onset diabetes remains poorly characterized. To preliminarily address this gap, we analyzed clinical data and subtype classifications within The Cancer Genome Atlas‐ Pancreatic Adenocarcinoma (TCGA‐PAAD) cohort. Our analysis found comparable rates of concurrent diabetes between basal and classical PDAC subtypes, with neither subtype demonstrating a significantly greater predisposition to new‐onset diabetes (Figure S9G, Supporting Information). Due to the relatively limited sample size in the TCGA cohort, these findings require further validation in larger prospective cohorts.

Emerging evidence indicates that secreted factors from diverse organs and tissues influence beta cell biology.^[^ ^100^ ^]^ MDK is expressed in multiple non‐pancreatic tissues interacting with PDAC, including adipose tissues and peripheral nerves.^[^ ^101^, ^102^ ^]^ This observation raises significant questions regarding whether crosstalk between the primary tumor and other MDK‐secreting tissues contributes to paraneoplastic diabetes in PDAC patients. Adipocytes, recognized for their potent secretory activity, engage in direct crosstalk with both PDAC cells and pancreatic islets, influencing tumor progression and beta cell function.^[^ ^103^, ^104^ ^]^ Crucially, MDK is detectable within adipose tissues and functions as an adipocyte‐derived secreted factor.^[^ ^101^ ^]^ We therefore hypothesize that adipocytes may directly impair islet function via MDK secretion, or indirectly exacerbate islet dysfunction by stimulating tumor cells to secrete elevated levels of MDK. Furthermore, PDAC is characterized by a dense, fibrotic stroma rich in neural cells.^[^ ^105^ ^]^ Xue et al. demonstrated that Schwann cells – the principal glial cells of peripheral nerves – migrate toward PDAC cells and enhance tumor migration via MDK secretion.^[^ ^102^ ^]^ This intricate tumor‐neural interplay leads us to propose additional mechanisms: Schwann cell‐derived MDK could directly compromise beta cell function. Alternatively, Schwann cells may secrete factors that upregulate MDK expression within PDAC cells, thereby amplifying paracrine signals detrimental to islets. While these proposed mechanisms are grounded in existing literature and observational data, rigorous experimental validations in future studies are needed to substantiate their specific contributions to paraneoplastic beta cell failure in PDAC.

This study has two key limitations that should be acknowledged. First, the unavailability of diabetes duration data precludes distinguishing PCAND from pre‐existing T2DM within our scRNA‐seq cohort. Consequently, comparative analyses of pathological mechanisms and biomarker explorations between these distinct diseases are not feasible. Future studies should incorporate diverse diabetic populations, such as those with PCAND, T2DM, and chronic pancreatitis‐associated diabetes, to better elucidate PCAND‐specific pathogenic mechanisms. Second, the restricted plasma sample size potentially diminishes statistical power and limits generalizability. Future investigations utilizing larger and multi‐center cohorts are warranted to substantiate the robustness of our conclusions.

**Figure 9:**
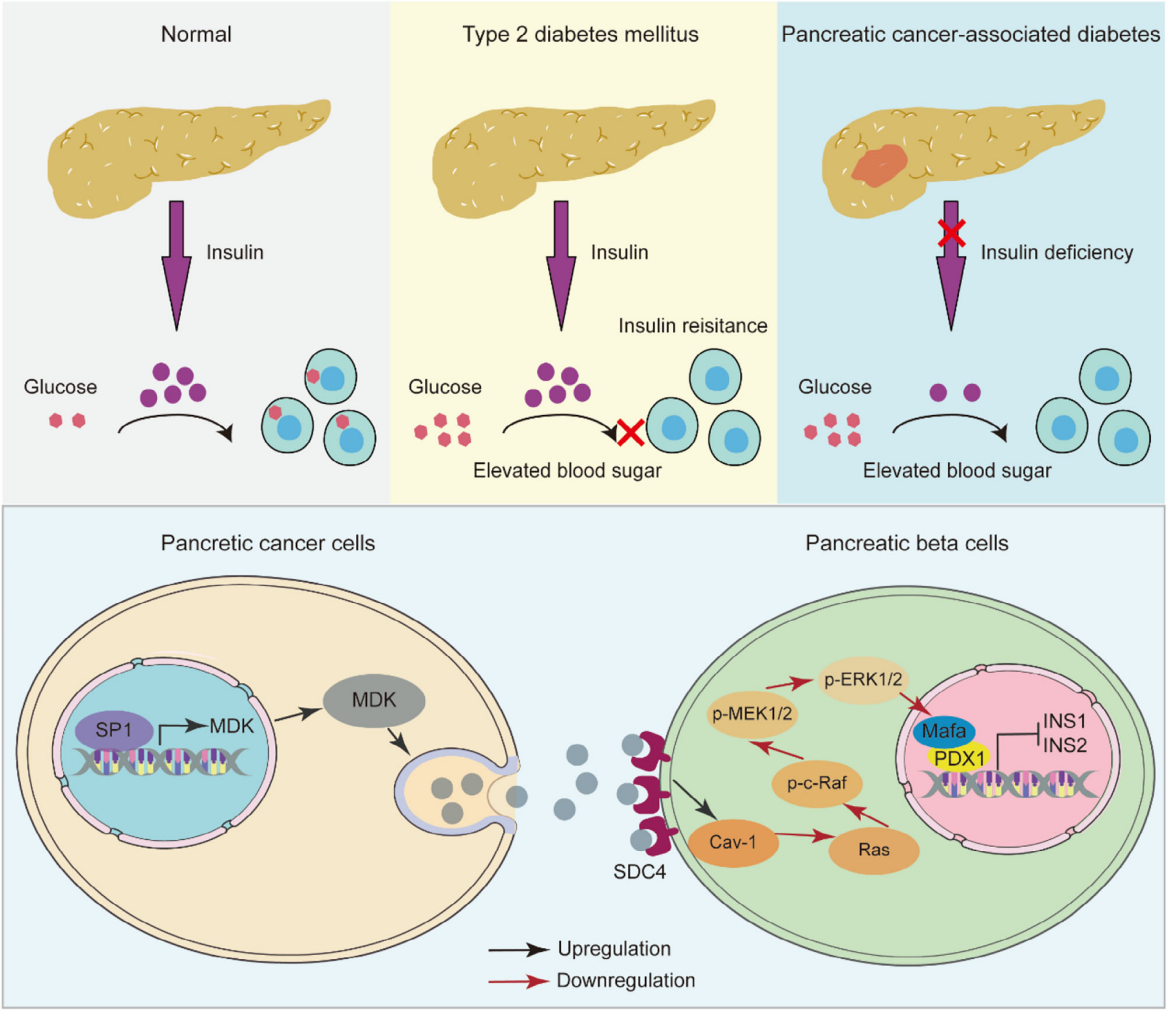
A schematic model of the SP1‐MDK‐SDC4‐Ras signaling axis in PCAND. Unlike T2DM, which is characterized primarily by insulin resistance, PCAND is characterized principally by insulin deficiency caused by tumor‐specific products. SP1 directly regulates MDK expression via binding its promoter in PDAC cells. MDK exerts inhibitory effects on beta cell function by directly binding SDC4 receptor and subsequently upregulating Cav1 to downregulate Ras signaling pathway in beta cells. Two TFs including MafA and Pdx1 are crucial for insulin synthesis and secretion. These two factors are in vitro substrates for ERK1/2, and their expression levels were inhibited by MDK‐SDC4 interactions, leading to insulin deficiency and the development of PCAND.

The involvement of the SP1‐MDK‐SDC4‐Ras signaling axis in the pathogenesis of PCAND is shown in **Figure** **9**. SP1 orchestrates MDK upregulation in PDAC cells. Subsequently, MDK secreted by tumor cells interacts with SDC4 on the surface of beta cells, thereby impairing beta cell function through inactivation of the Ras signaling pathway. Our study reveals the pivotal role of MDK‐SDC4 interactions in pancreatic cancer‐induced paraneoplastic diabetes, providing fresh insights into the pathogenesis of PCAND. Additionally, it is noteworthy that plasma MDK levels were markedly elevated in patients with PCAND compared to those in patients with T2DM, suggesting that plasma MDK holds promise as a potential biomarker for the early screening of PDAC in the new‐onset diabetes population.

## Conflict of Interest

The authors declare no conflict of interest.

## Author Contributions

Z.F., J.L., and C.L., These authors contributed equal to this paper.Y.W. and Z.F. conceived and designed the study. Z.F., J.L., C.L., and H.Y. conducted most of the experiments and interpreted the results. J.L., J.G., and X.L. contributed to the acquisition of clinical samples. Z.F. and H.T. contributed to the bioinformatic analysis. Z.F., H.Y., and H.T. contributed to the in vivo studies. Z.F. drafted the original manuscript. Y.W. and X.L. gave professional comments on the study and revised the manuscript. All authors have approved the final manuscript.

## Supporting information



Supporting Information

Supplemental Table 1

## Data Availability

The data that support the findings of this study are available from the corresponding author upon reasonable request.
