# Supplementary material for: Unraveling the Role of MDK‐SDC4 Interaction in Pancreatic Cancer‐Associated New‐Onset Diabetes by Single‐Cell Transcriptomic Analysis
Source: Adv Sci (Weinh). 2025 Jul 25;12(39):e09987. doi: 10.1002/advs.202409987 (PMC12533153; doi:10.1002/advs.202409987)
Supplement: Supplementary file 1 — Supporting Information [file ADVS-12-e09987-s002.docx]

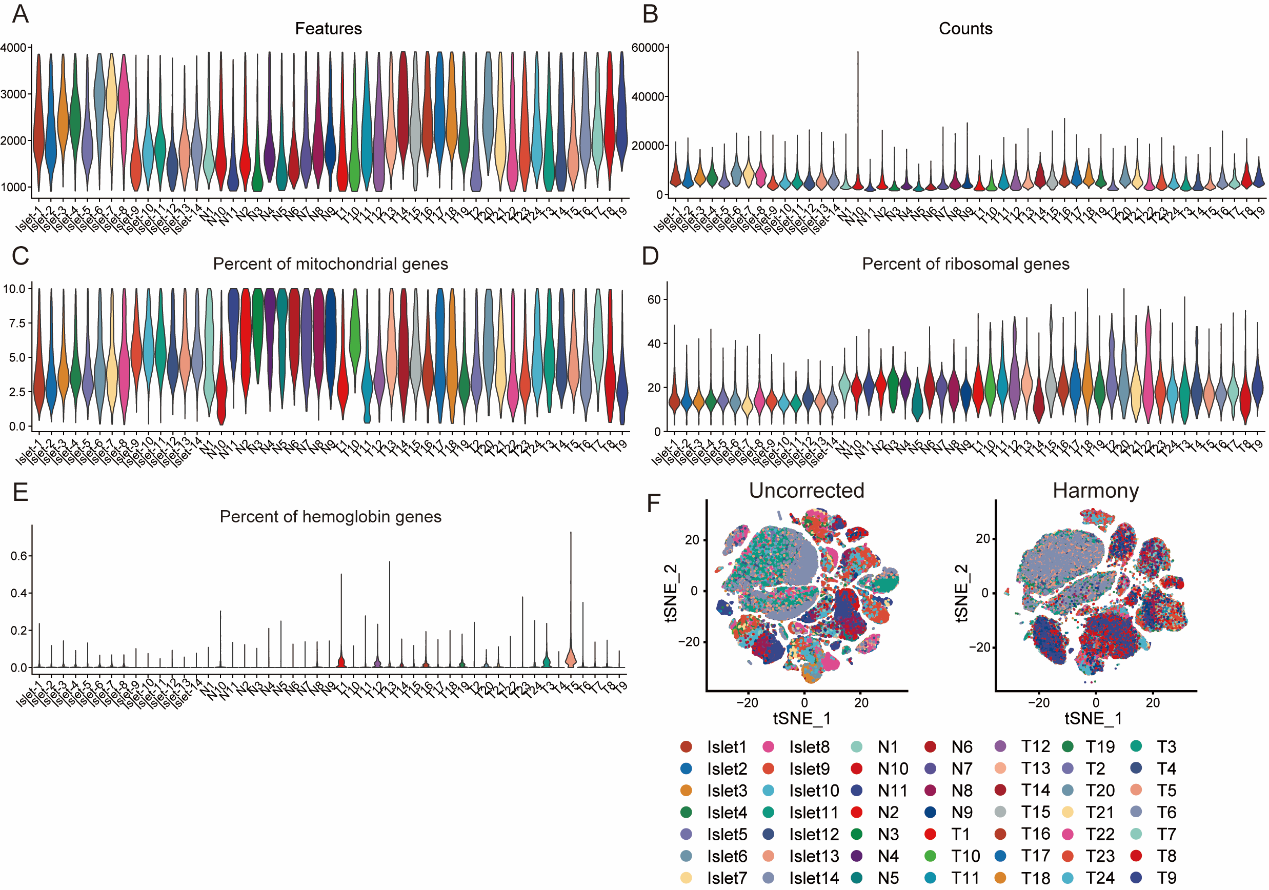


**Figure S1. Quality control of single-cell RNA-seq data.** (A) Violin plots showing the number of features detected in different samples. (B) Violin plots showing the number of counts detected in different samples. (C) Violin plots showing the percentage of mitochondrial genes detected in different samples. (D) Violin plots showing the percentage of ribosomal genes detected in different samples. (E) Violin plots showing the percentage of hemoglobin genes detected in different samples. (F) t-SNE plots visualizing cells from data after Harmony-mediated batch correction versus uncorrected data.


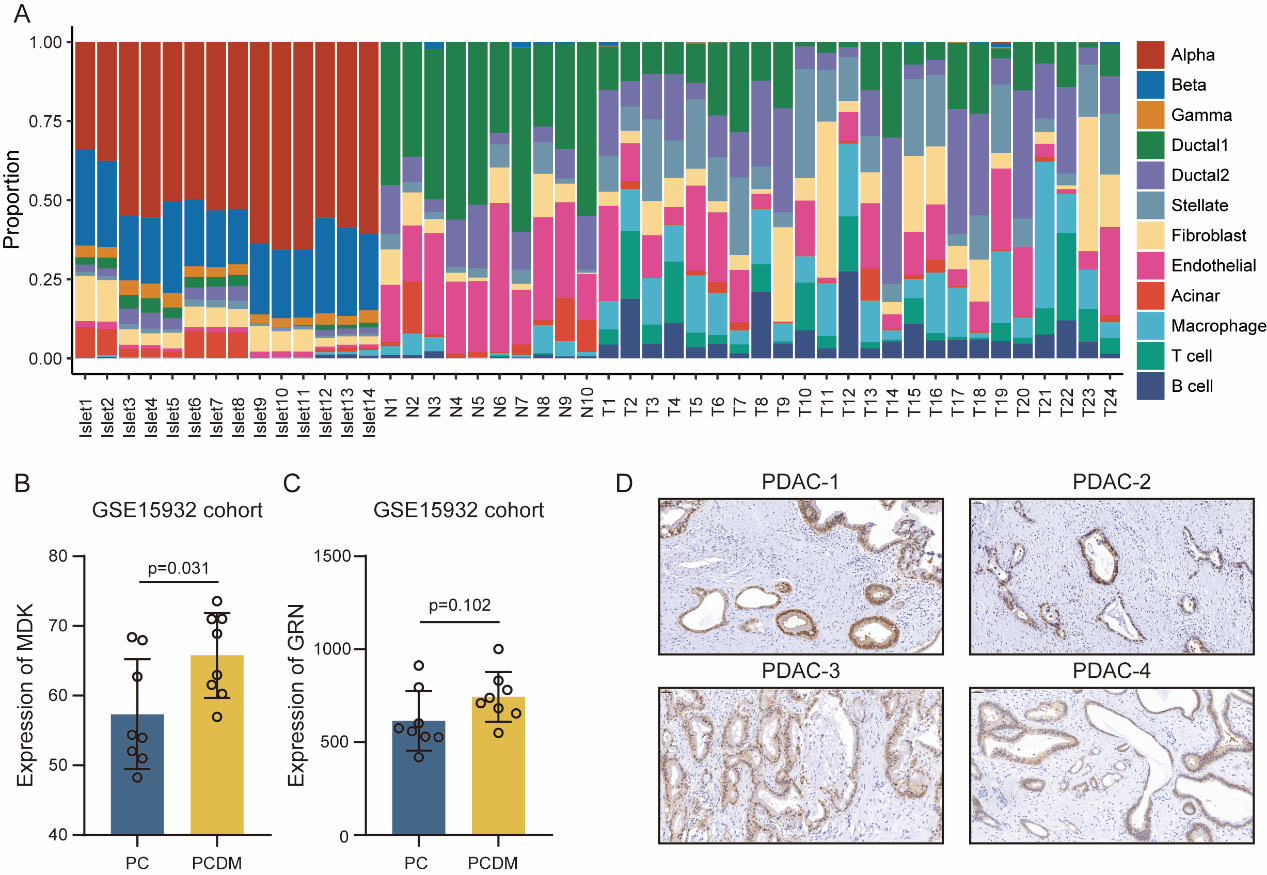


**Figure S2. Relative cell enrichment, MDK expression in GSE15932 cohort and MDK protein expression in PDAC tissues.** (A) Bar plot showing the proportion of each cell type in 24 PDAC patients, 11 non-PDAC control patients and 14 normal human islets. (B, C) Expression of MDK (B) and GRN (C) in peripheral blood samples from 8 PDAC patients without diabetes and 8 PDAC patients with diabetes based on GSE15932 cohort. (D) Representative IHC staining images showing MDK expression in tumor and stromal components of PDAC tissues.


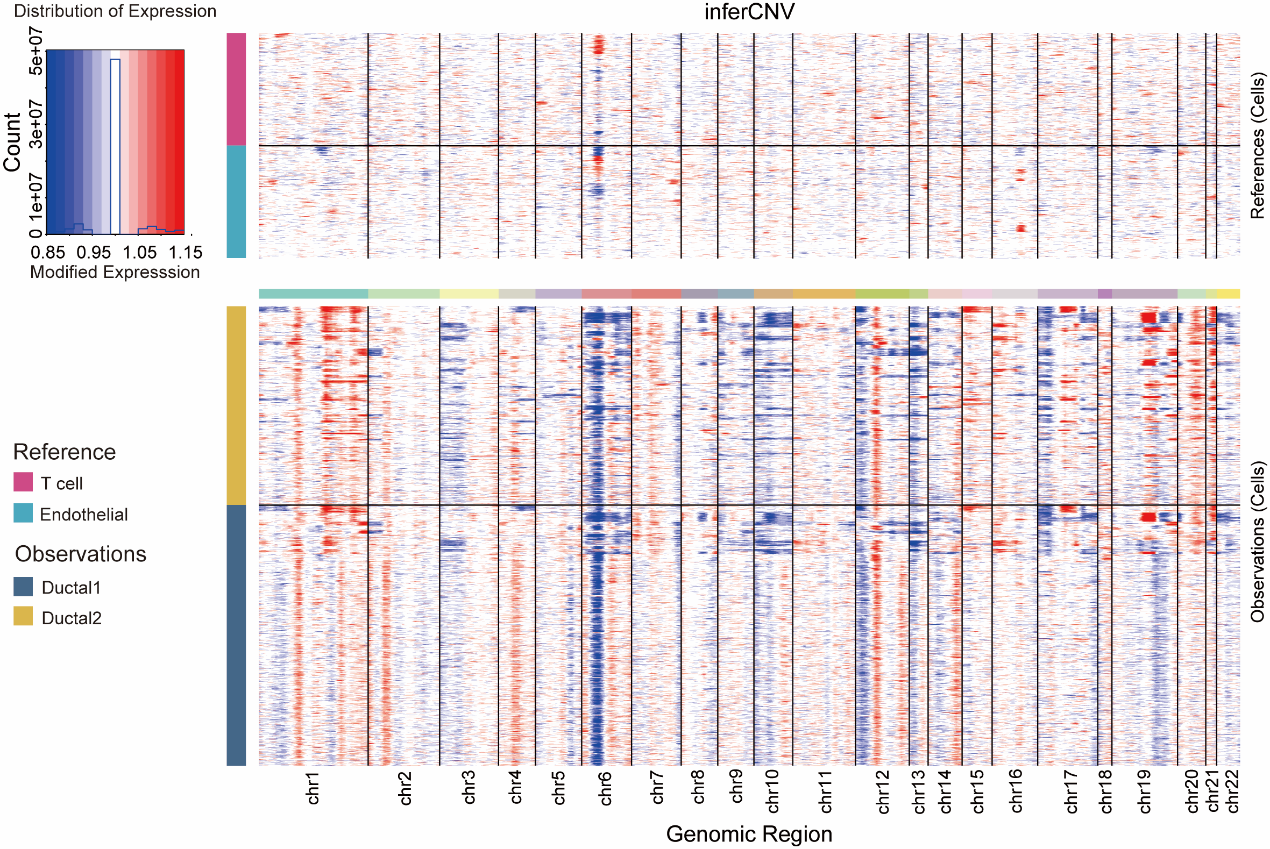


**Figure S3. CNV prediction by inferCNV in each cell type.**

**
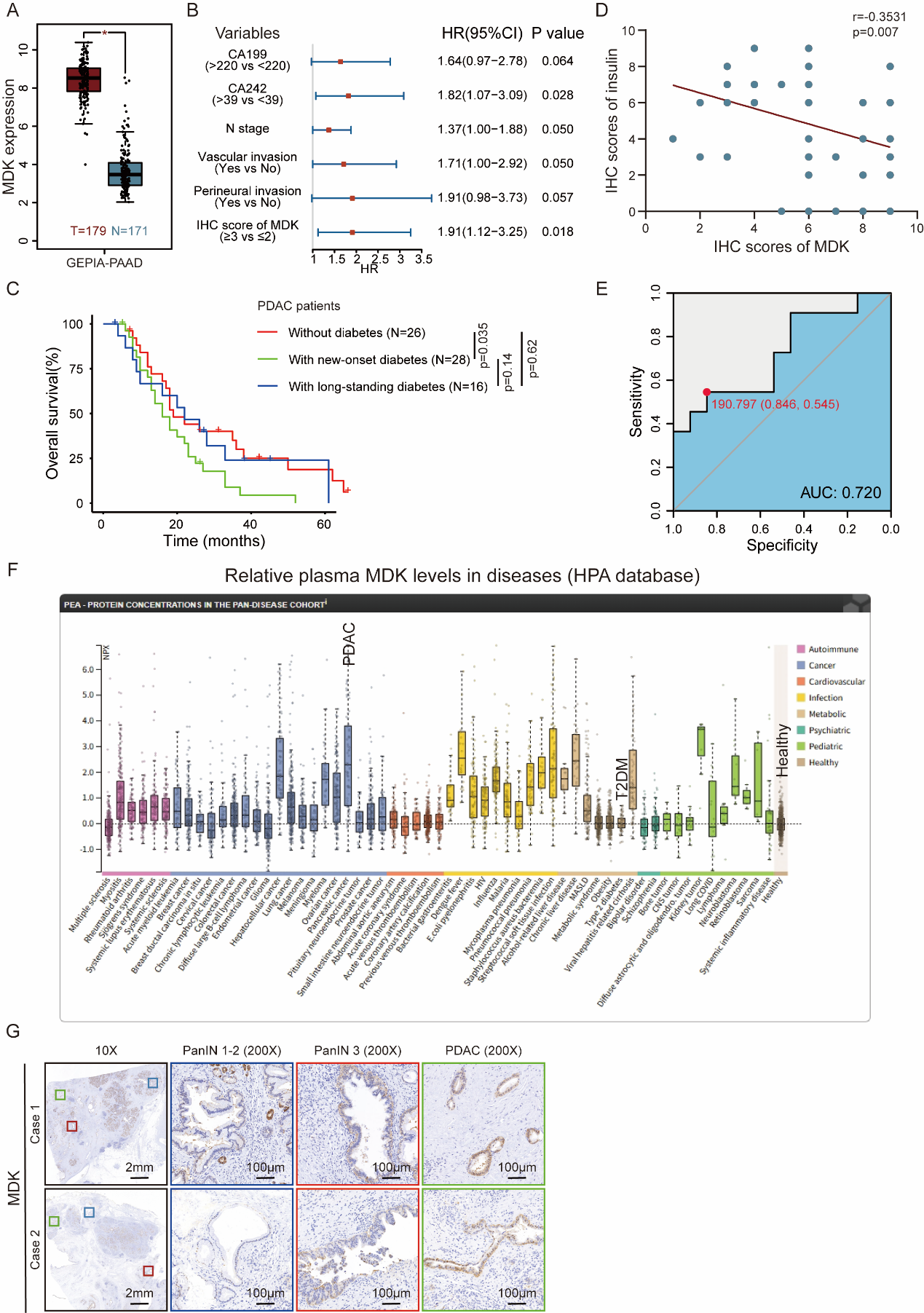
**

**Figure S4. Expression of MDK.** (A) The mRNA levels of MDK in pancreatic adenocarcinoma and normal pancreases from the GEPIA database. (B) Univariate Cox regression analyses of the survival of patients with PDAC. (C) Survival differences between PDAC without diabetes, PDAC with now-onset diabetes and PDAC with long-standing diabetes in our cohort. (D) Correlation between MDK expression levels in tumor tissues and insulin levels in adjacent islet tissues in 57 PDAC sections. (E) ROC curves for plasma MDK levels to distinguish PCAND from T2DM. The red dot represents the optimal cut-off point. (F) Relative plasma concentrations of MDK protein in blood samples from patients with different types of disease were measured by proximity extension assay. This picture is downloaded from the Human Protein Altas portal (https://www.proteinatlas.org/ENSG00000110492-MDK/blood). (G) The representative IHC staining images of MDK in low-grade PanIN (PanIN 1-2, blue), high-grade PanIN (PanIN 3, red) and PDAC (green) lesions.


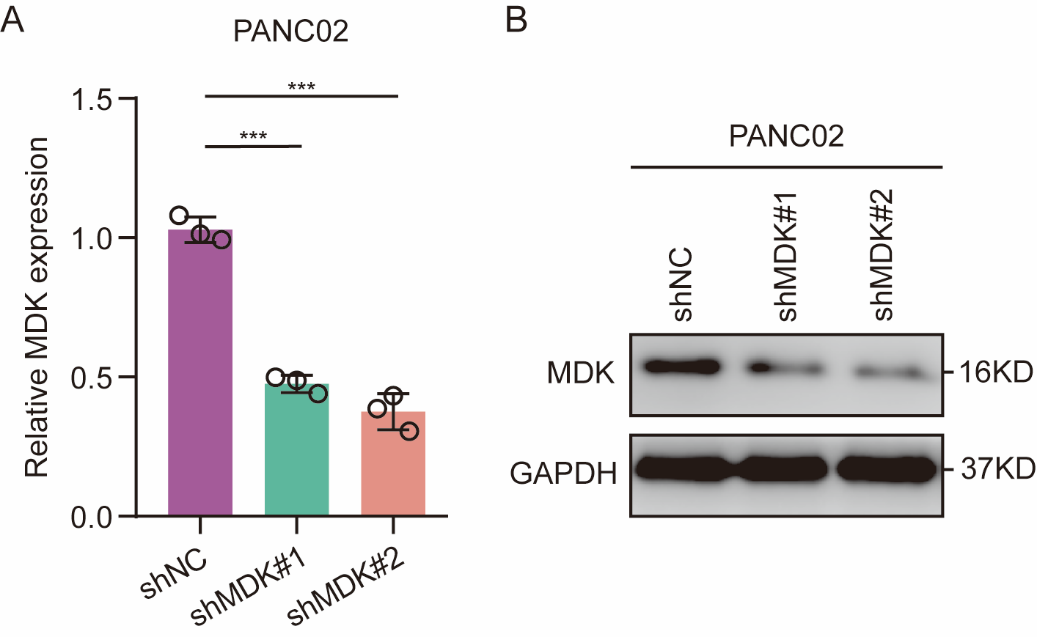


**Figure S5. Successful knockdown of MDK in PANC02 cells.** (A) The relative mRNA levels of MDK in PANC02 cells were measured by RT-qPCR. (B) The protein levels of MDK in PANC02 cells were detected by western blotting. ***, P < 0.001, means ± SD was shown. Student’s t-test analysis was used for comparison between two groups.

**
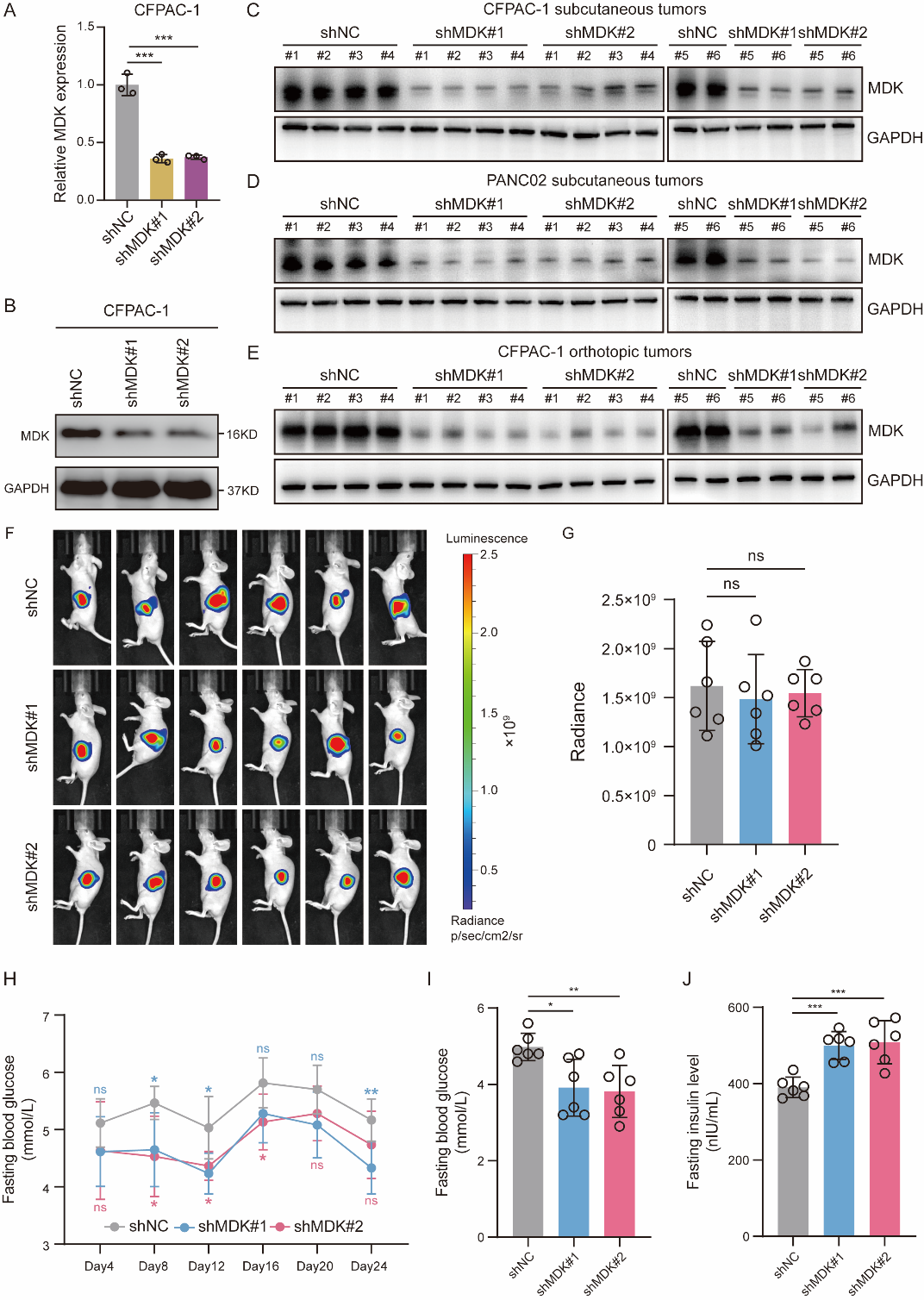
**

**Figure S6. MDK damages beta cell function in vivo.** (A) RT-qPCR analysis of MDK mRNA levels in CFPAC-1 cells. (B) Western blotting analysis of MDK protein levels in CFPAC-1 cells. (C-E) Western blotting detection of MDK expression in CFPAC-1 subcutaneous tumors (C), PANC02 subcutaneous tumors (D), and CFPAC-1 orthotopic tumors (E). (F) Bioluminescence imaging visualizing the growth of orthotopic xenografts formed by luciferase-expressing CFAPC-1 cells with or without MDK knockdown. (G) Summary of average radiance from the pancreas area for each group. (H) Fasting blood glucose (fasting 6 hours before sampling) monitoring was performed every 4 days after orthotopic transplantation in nude mice. (I) Detection of fasting blood glucose (fasting 24 hours before sampling) in nude mice before sacrifice. (J) Measurement of fasting insulin levels (fasting 24 hours before sampling) in nude mice before sacrifice. ns, not significant; *, P < 0.05; **, P < 0.01; ***, P < 0.001, means ± SD was shown. Student’s t-test analysis was used for comparison between two groups.

**
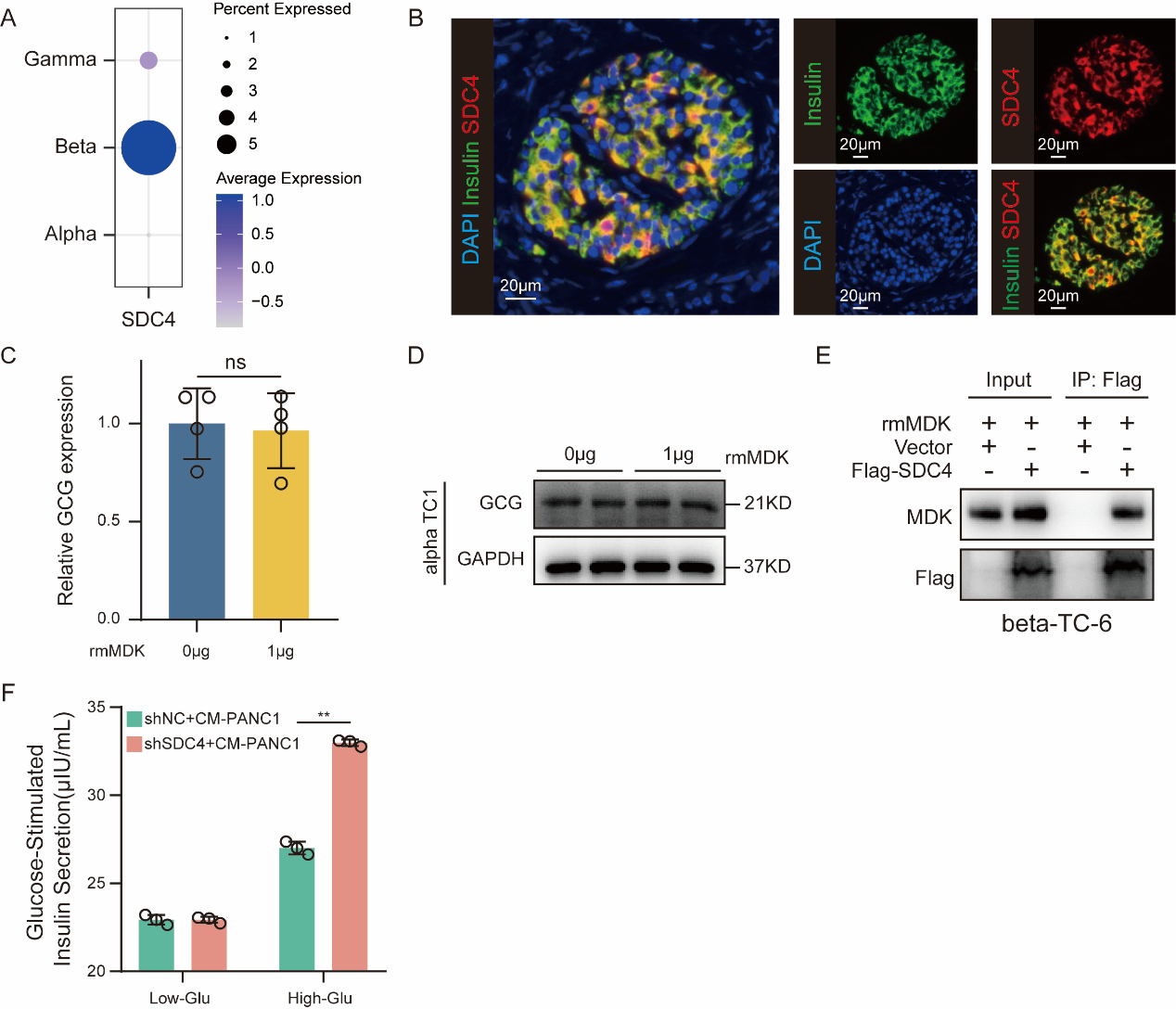
**

**Figure S7. SDC4 expression in pancreatic islets.** (A) Dot plot showing the expression of SDC4 across the cell types identified in pancreatic islets. (B) Representative images of multiplex immunofluorescence co-staining for SDC4 (red) and insulin (green) in resected PDAC tissues. (C) The relative mRNA levels of GCG (Glucagon) in alpha cells (alpha TC1) treated with or without recombinant mouse MDK protein (rmMDK). (D) The protein expression levels of GCG in alpha cells treated with or without rmMDK. GAPDH was used as the loading control. (E) The co-IP assay analyzed the interaction between SDC4 and MDK. (F) Low and high glucose-stimulated insulin secretion levels in the indicated beta cells treated with conditioned mediums from PANC1 cells. ns, not significant; *, P < 0.05; **, P < 0.01; ***, P < 0.001, means ± SD was shown. Student’s t-test analysis was used for comparison between two groups.


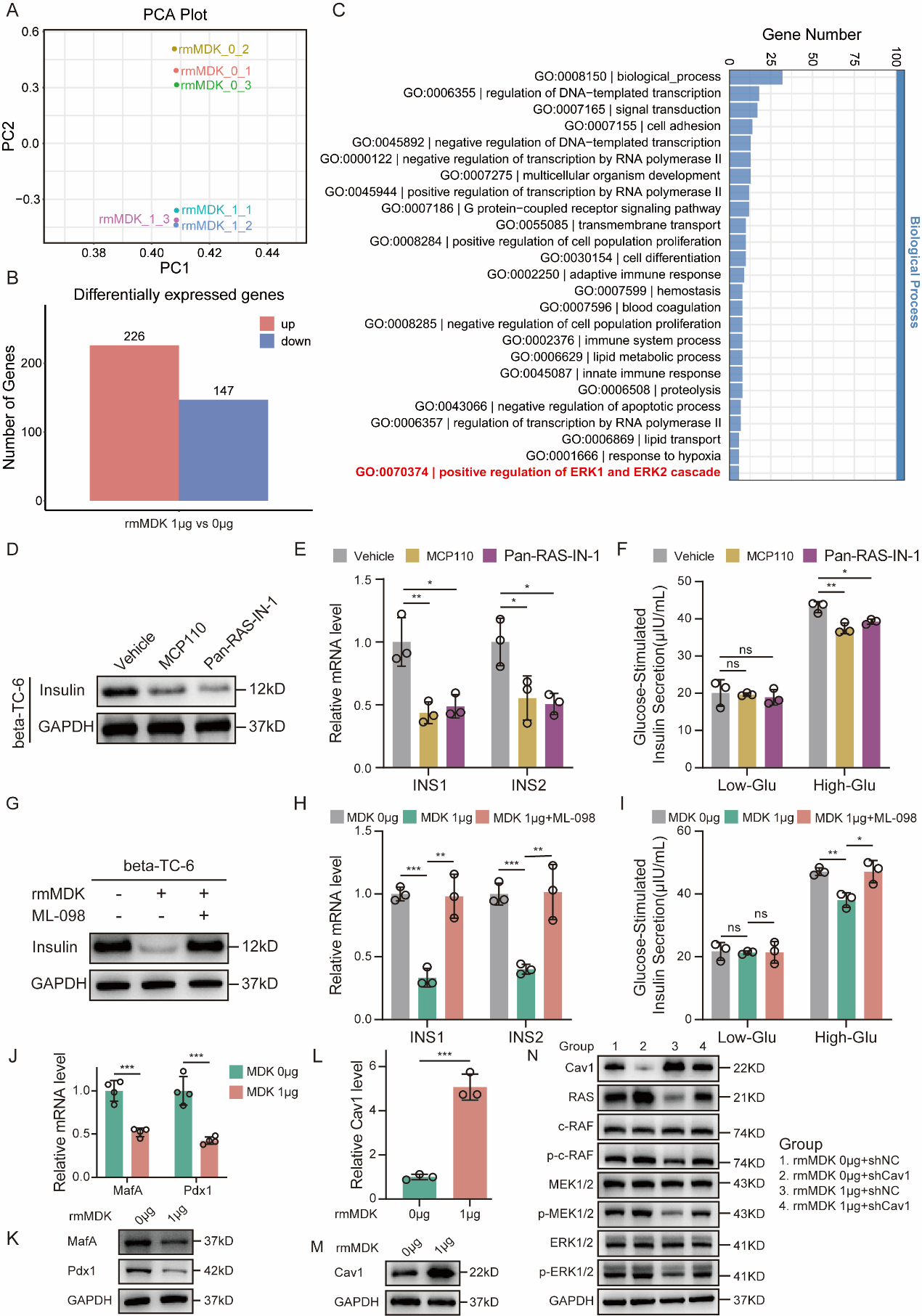


**Figure S8. MDK-SDC4 interaction impairs beta cell function by inhibiting Ras signaling pathway.** (A) Principal component analysis of rmMDK-treated and rmMDK-untreated beta-TC-6 cells. (B) Bar plot showing differentially expressed genes between rmMDK-treated and rmMDK-untreated beta cells. (C) Bar plot of GO-Biological Process enrichment analysis. (D) The protein expression levels of insulin in beta-TC-6 cells treated with Ras inhibitors were detected by western blotting. (E) The relative mRNA levels of INS1 and INS2 in beta cells treated with Ras inhibitors were measured by RT-qPCR. (F) Low and high glucose-stimulated insulin secretion levels in beta cells treated with indicated agents. (G) The protein expression levels of insulin in beta-TC-6 cells treated with indicated agents. (H) The relative mRNA levels of INS1 and INS2 in beta cells treated with indicated agents. (I) Low and high glucose-stimulated insulin secretion levels in beta cells treated with indicated agents. (J) The mRNA levels of MafA and Pdx1 in beta-TC-6 cells treated with or without rmMDK. (K) The protein levels of MafA and Pdx1 in beta-TC-6 cells treated with or without rmMDK. (L) The relative mRNA levels of Cav1 in beta-TC-6 cells treated with or without rmMDK. (M) The protein expression levels of Cav1 in beta-TC-6 cells treated with or without rmMDK. (N) Western blotting analysis of the Ras/Raf/MEK/ERK signaling pathway in different groups. ns, not significant; *, P < 0.05; **, P < 0.01; ***, P < 0.001, means ± SD was shown. Student’s t-test analysis was used for comparison between two groups.


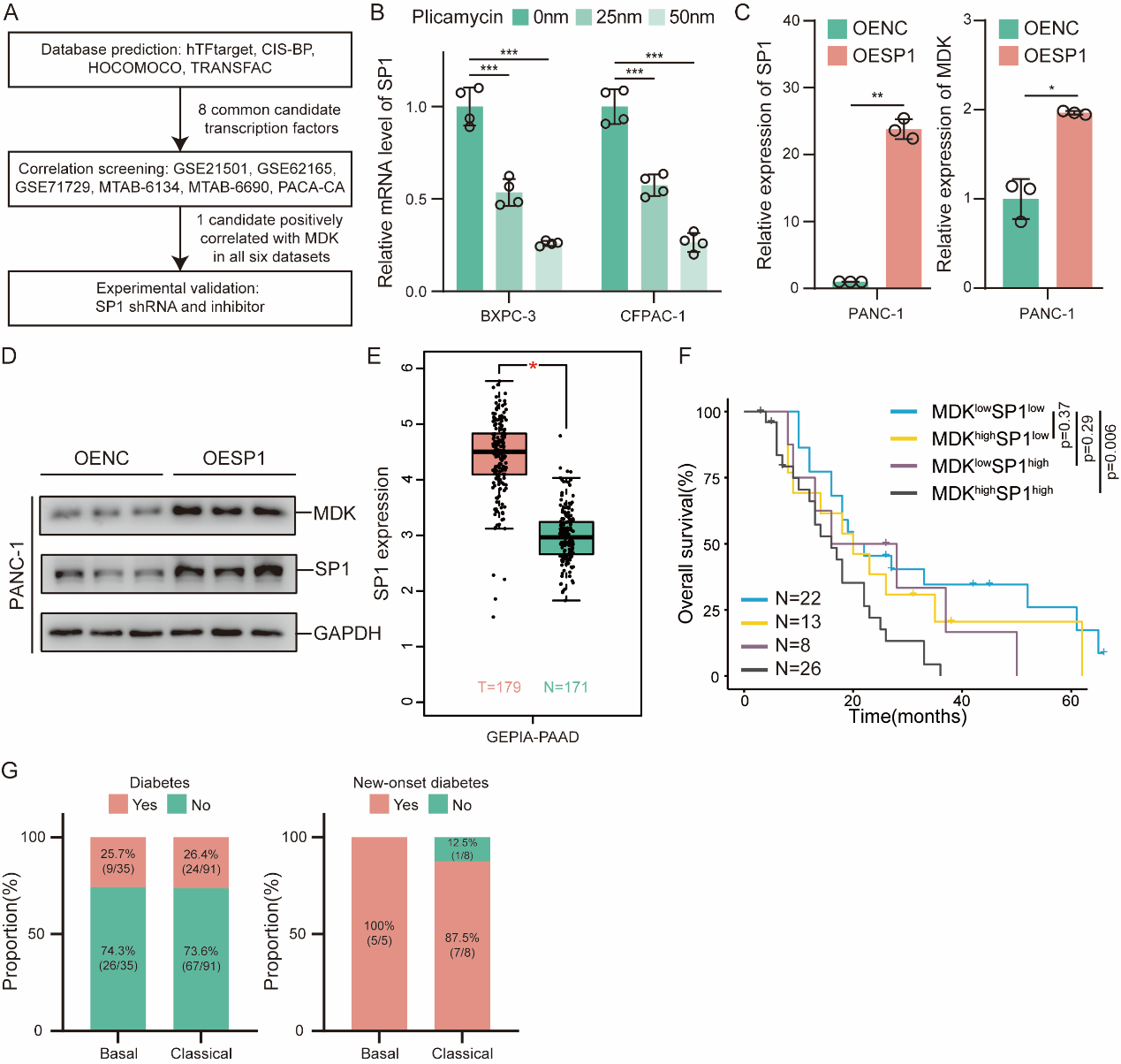


**Figure S9. SP1 transcriptionally activates MDK in PDAC cells.** (A) Workflow of identifying upstream transcriptional regulators of MDK. (B) RT-qPCR analysis of SP1 mRNA levels in BxPC-3 and CFPAC-1 cells treated with Plicamycin. (C) RT-qPCR analysis of SP1 and MDK mRNA levels in PANC1 cells. (D) Western blotting analysis of SP1 and MDK protein levels in PANC1 cells. (E) The mRNA levels of SP1 in pancreatic adenocarcinoma and normal pancreases from the GEPIA database. (F) Kaplan–Meier survival analyses of overall survival according to the expression of MDK, SP1, or the combined group in PDAC patients using log-rank test. (G) Left panel: Proportions of PDAC patients with or without comorbid diabetes across transcriptomic subtypes. Right panel: Proportions of PDAC patients with new-onset diabetes (onset ≤2 years before PDAC diagnosis) or with pre-existing diabetes (onset >3 years before PDAC diagnosis) across transcriptomic subtypes. Comparative analyses utilized PDAC patients from the TCGA-PAAD cohort. *, P < 0.05; **, P < 0.01; ***, P < 0.001, means ± SD was shown. Student’s t-test analysis was used for comparison between two groups.
